# A Quartet of PIF bHLH Factors Provides a Transcriptionally Centered Signaling Hub That Regulates Seedling Morphogenesis through Differential Expression-Patterning of Shared Target Genes in *Arabidopsis*

**DOI:** 10.1371/journal.pgen.1003244

**Published:** 2013-01-31

**Authors:** Yu Zhang, Oleg Mayba, Anne Pfeiffer, Hui Shi, James M. Tepperman, Terence P. Speed, Peter H. Quail

**Affiliations:** 1 Department of Plant and Microbial Biology, University of California Berkeley, Berkeley, California, United States of America; 2 Plant Gene Expression Center, Agriculture Research Service, U.S. Department of Agriculture, Albany, California, United States of America; 3 Department of Statistics, University of California Berkeley, Berkeley, California, United States of America; The University of North Carolina at Chapel Hill, United States of America

## Abstract

Dark-grown seedlings exhibit skotomorphogenic development. Genetic and molecular evidence indicates that a quartet of *Arabidopsis* Phytochrome (phy)-Interacting bHLH Factors (PIF1, 3, 4, and 5) are critically necessary to maintaining this developmental state and that light activation of phy induces a switch to photomorphogenic development by inducing rapid degradation of the PIFs. Here, using integrated ChIP–seq and RNA–seq analyses, we have identified genes that are direct targets of PIF3 transcriptional regulation, exerted by sequence-specific binding to G-box (CACGTG) or PBE-box (CACATG) motifs in the target promoters genome-wide. In addition, expression analysis of selected genes in this set, in all triple *pif*-mutant combinations, provides evidence that the PIF quartet members collaborate to generate an expression pattern that is the product of a mosaic of differential transcriptional responsiveness of individual genes to the different PIFs and of differential regulatory activity of individual PIFs toward the different genes. Together with prior evidence that all four PIFs can bind to G-boxes, the data suggest that this collective activity may be exerted via shared occupancy of binding sites in target promoters.

## Author Summary

An important issue in understanding mechanisms of eukaryotic transcriptional regulation is how members of large transcription-factor families, with conserved DNA–binding domains (such as the 162-member *Arabidopsis* bHLH family), discriminate between target genes. However, the specific question of whether, and to what extent, closely related sub-family members, with potential overlapping functional redundancy (like the quartet of Phytochrome (phy)-Interacting bHLH transcription Factors (PIF1, 3, 4, and 5) studied here), share regulation of target genes through shared binding to promoter-localized consensus motifs does not appear to have been widely investigated. Here, using ChIP–seq analysis, we have identified genes that bind PIF3 to conserved, sequence-specific sites in their promoters; and, using RNA–seq, we have identified those genes displaying altered expression in various *pif* mutants. Integration of these data identifies those genes that are likely direct targets of transcriptional regulation by PIF3. Our data suggest that the PIF quartet members share directly in transcriptional activation of numerous target genes, potentially via redundant promoter occupancy, in a manner that varies quantitatively from gene to gene. This finding suggests that these PIFs function collectively as a signaling hub, selectively partitioning common upstream signals from light-activated phys at the transcriptional-network interface.

## Introduction

A key component of the successful colonization of land by terrestrial flowering plants was the evolution of a developmental strategy termed skotomorphogenesis (etiolated growth). This strategy enabled post-germinative seedlings emerging from buried seed to grow heterotrophically, on seed reserves, rapidly upwards through the subterranean darkness to the soil surface. Coupled with this was the evolution of a photosensory mechanism to trigger a switch to autotrophic, photomorphogenic (deetiolated) development upon emergence into sunlight.

Genetic evidence indicates that a small subfamily of basic helix-loop-helix (bHLH) transcription factors, termed PIFs (for Phytochrome (phy)-Interacting Factors) are centrally critical to the promotion of such skotomorphogenic development in dark-grown seedlings [1]. A quadruple *pif* mutant (*pifq*), lacking PIF-family members PIF1, PIF3, PIF4 and PIF5 (termed the PIF quartet), displays morphogenic development in total darkness that strongly phenocopies that of normal light-grown seedlings [2], [3]. This observation establishes that these factors act constitutively to promote skotomorphogenic development and that their absence induces the switch to photomorphogenic development. All four quartet members have been shown individually to bind preferentially to a core G-box DNA-sequence motif (CACGTG) (a variant of the canonical E-box motif (CANNTG)) [4]–[9], and to function as transcriptional activators in transfection or heterologous systems [4]–[7], [10]. Because monogenic mutants at each of these loci have no, or minimal, visible effects on skotomorphogenesis, and the various double and triple *pif*-mutant combinations progressively exhibit increasingly photomorphogenic phenotypes in darkness, it appears that the PIF quartet members act with partially additive or overlapping redundancy to drive the skotomorphogenic pathway [2], [3], [11]–[13].

The phy family of sensory photoreceptors (especially phyA and phyB) has a central role in inducing the switch from skotomorphogenic to photomorphogenic development (deetiolation) in response to initial exposure of dark-grown seedlings to light [1], [14], [15]. The existing evidence indicates that this is achieved in large part by rapid phy-triggered degradation of the PIF proteins. The mechanism underlying this process involves the rapid, light-induced translocation of the activated (Pfr) conformer of the phy molecule from the cytoplasm into the nucleus, where it physically interacts with PIF-quartet members. This interaction induces phosphorylation of the PIF proteins which in turn triggers ubiquitylation and proteolytic degradation of the transcription factors (half-lives of 5–20 min) via the proteasome system. The altered transcriptional landscape resulting from the consequent robust reduction in steady-state abundance of these factors is the major driving force in the switch from heterotrophic to autotrophic development inherent in the deetiolation process.

A limited number of transcriptome analyses, using Affymetrix ATH1 microarrays, aimed at identifying genes regulated by the phy-PIF signaling pathway during deetiolation have been reported [3], [12], [16]–[19]. The data show that 80% of the genes that display altered expression in the *pifq* mutant in the dark are normally altered by prolonged light in fully deetiolated wild-type (WT) seedlings [17], but that only a relatively small fraction of these are misexpressed in dark-grown *pif1* [19], *pif3* [16]–[18] and *pif4pif5* mutants [12]. These results affirm the central collective regulatory function of these four PIFs in regulating the overall transcriptional network that drives the developmental switch from skotomorphogenesis to photomorphogenesis, and provide initial indications of functional redundancy at the gene expression level. These genes could be either direct or indirect targets of PIF transcriptional regulatory activity [20]. Identification of those genes that respond rapidly (within 1 h) to initial light exposure has defined a subset of PIF-regulated genes that are likely to be enriched for loci that are directly transcriptionally regulated by the PIF-quartet proteins [17]. PIF-regulated genes that conversely respond rapidly to vegetative shade in fully-green, light-grown plants have also been identified by microarray-based expression profiling [11], [21]. It is notable that these early-response genes are enriched for transcription-factor-encoding loci, suggesting a potential hierarchal network that drives a transcriptional cascade. However, rapid responsiveness alone obviously does not establish that transcriptional regulation is direct.

The advent of ChIP-chip and ChIP-seq technology has provided the opportunity to identify genes that contain binding sites for transcription factors of interest, on a genome-wide scale [20], [22], [23]. When combined with full transcriptome analysis, the data provide identification of genes that are direct targets of transcriptional regulation by the factor(s) under study. A number of such studies have recently been reported for a diversity of factors in *Arabidopsis*, using either ChIP-chip or ChIP-seq analysis of factor binding sites, coupled predominantly with Affymetrix ATH1 microarrays (representing about 80% of the protein-coding genes in the genome) for expression analysis [21], [23]–[29]. These data have begun to provide insight into the complexity of the transcriptional networks that coordinate a variety of the fundamental processes underlying plant growth and development.

Despite these advances, the use of the ATH1 microarray for expression analysis in many of these studies means that important expression changes in genes not present on this array might have been missed. In addition, the question of whether, and to what extent, closely related transcription-factor family members, such as the PIF quartet, with apparently shared DNA-target-sequence specificity, contribute toward the transcriptional regulation of common target genes does not appear to have been addressed in many existing studies of eukaryotic systems [27], [30]–[34], although a recent report by Hornitschek et al shows differential binding of recombinant PIF4 and PIF5 to various E-box variants in vitro using protein-binding microarrays, as well as shared binding *in vivo* to four selected promoters using ChIP-PCR analysis [21]. Here, using ChIP-seq analysis, we have identified PIF3-binding sites, genome wide, and, in parallel, using RNA-seq analysis of selected *pif*-mutant lines, we have defined the genes regulated by PIF3, genome-wide, in dark-grown seedlings. By merging these datasets, we have identified those genes whose expression is, at least partially, directly regulated by promoter-bound PIF3. In addition, by profiling the expression of a selected subset of these direct PIF3-targets in multiple additional *pif*-mutant combinations, we have addressed the question of whether PIF1, 3, 4 and 5 display qualitative and/or quantitative functional divergence in regulating shared target genes.

## Results

### ChIP–seq–based identification of PIF3-binding sites

**Figure 1:**
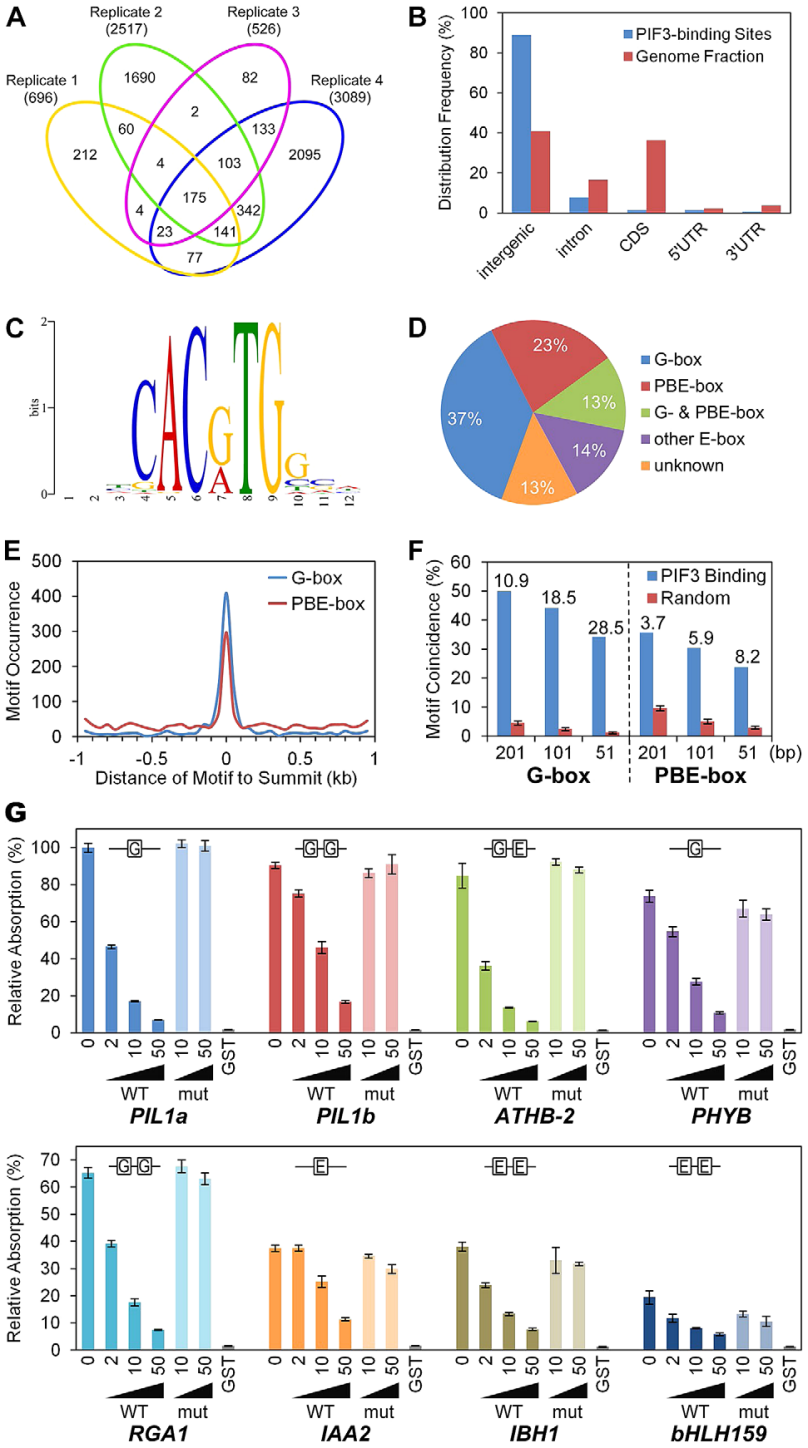
Genome-wide identification of PIF3-binding sites and motifs. (A) Venn diagram depicting total numbers (parentheses) and reproducible presence (overlapping sectors) of statistically significant PIF3-binding peaks in ChIP-seq analysis of four biological replicates (Venn ovals) of dark-grown seedlings. (B) Relative binding-peak distribution across genomic regions. (C) MEME motif search identifies two dominant PIF3-binding motifs, defined as G-box (CACGTG) and PBE-box (CACATG) motifs. (D) Percentage of PIF3 binding sites containing designated motifs. Other E-box: Variants of E-box (CANNTG) motif other than G- or PBE-box. Unknown: Unknown and/or non-statistically-overrepresented motif. (E) Distribution of the G- and PBE-box motifs in the 1 kb regions surrounding the PIF3-binding peak-summits. (F) G- and PBE-box-motif coincidence with PIF3-binding peaks (% within 201, 101, and 51 bp centered at the peak-summits) is significantly higher than in other random genomic regions of the same size. Internal numbers indicate the relative fold motif-enrichment at PIF3 binding-sites. Error bars represent the standard deviation of 100 random simulations. (G) DPI-ELISA assays of *in vitro* binding of recombinant GST-PIF3 to the G- and PBE-box motifs. Binding activity (Relative Absorbance) for each DNA probe is expressed as a percentage of each reaction relative to GST-PIF3 binding to the *PIL1a* WT probe. Data represent the mean of independent duplicates +/− SEM. WT: wild-type competitor probes. mut: competitor probes mutated at the G-box and PBE-box motifs. GST: GST negative-control binding to the biotinylated WT probes.

Two-day-old dark-grown wild-type (WT) and MYC-epitope-tagged-PIF3 (P3M)-expressing, *pif3-3* null-mutant seedlings were used for ChIP-seq analysis. DNA prepared from MYC-antibody-generated immunoprecipitates from four independent biological replicates of each genotype was subjected to high-throughput sequencing. Statistically-significant binding peaks were defined by comparing the parallel P3M and WT ChIP samples within each replicate using the MACS algorithm [35]. Replicate-specific peaks (Table S1) were defined as reproducible if they were identified at the same genomic location in two or more biological replicates (overlapping Venn sectors in Figure 1A; also Table S2). For each reproducible peak, we assigned a common summit as the mean of the individual replicate-specific summits.

This analysis identified 1064 reproducible peaks which form our “high-confidence” set of PIF3-binding sites (Table S2). These sites are evenly distributed on the five chromosomes and 89% are located in intergenic regions (Figure 1B). In ChIP-qPCR validation assays, all but 1 of the 38 tested regions exhibited strong binding enrichment in the P3M samples compared to the WT controls (Figure S1), indicating a low false positive rate for our ChIP-seq procedure.

### G-box and PBE-box motifs dominate PIF3-binding DNA sequences

Using the MEME program [36], we performed *de novo* motif discovery on the +/−100 bp regions surrounding the 1064 “high-confidence” PIF3 binding-peak summits described above. Two E-box (CANNTG) variants were identified as statistically overrepresented motifs within these PIF3-binding regions (Figure 1C). The CACGTG (‘G-box’) variant is well-established as a preferred PIF-binding motif [4]–[9]. By contrast, the CACATG variant is previously undescribed as a PIF3-binding motif, although PIF1 [37] and, recently, PIF4 [21] have been reported to bind. We conclude that this variant is a strong candidate for being a general alternative binding motif for PIF3 across the genome, and define it, therefore, as the PIF-binding E-box (PBE-box). The relative distribution of these two motifs across the 1064 PIF3 binding-sites is summarized in Figure 1D. A majority (73%) of the sites contain one or both motifs (G-box 50% and PBE-box 36%, with 13% overlap) within the 200-bp window.

A broader analysis shows that 64% of the G-box and 30% of the PBE-box motifs present in the 2 kb windows surrounding the PIF3-binding summits cluster within the designated 200-bp binding sites (Figure 1E). Similarly, both motifs are strongly enriched in these 200-bp windows compared to random 200-bp genome segments, and this enrichment increases toward the PIF3-binding summit (Figure 1F). These data establish the highly significant coincidence between PIF3 and these two specific *cis*-elements.

To examine the potential direct interaction of PIF3 with the newly-identified PBE-box compared to that of the G-box, we performed DNA-Protein-Interaction (DPI)-ELISA [38]. We tested the binding of PIF3 to several G-box- (*PIL1*, *PHYB*, and *RGA1*) or PBE-box- (*IAA2*, *IBH1*, and *AT4G30410*) containing probes generated from various genomic PIF3-binding sites identified in the ChIP-seq analysis. Figure 1G shows that recombinant PIF3 binds sequence-specifically to all G-box- and PBE-box-containing probes, although the apparent affinity for the G-box seems overall to be higher than for the E-box. An EMSA analysis showed similar results (Figure S2). These *in vitro* binding-assay data indicate that the coincidence of PIF3-binding sites with the G-box or PBE-box motif in the ChIP-seq assay likely results from their direct interaction *in vivo*, and that the PBE-box is indeed another sequence-specific PIF-binding, E-box variant genome wide. Because all of the PIF3-binding sites tested by ChIP-qPCR in Figure S1 contain coincident G- or PBE-box motifs, these data validate the *in vivo*-binding of PIF3 to these motifs.

The binding of PIF3 to the *ATHB-2* probe, which contains one G-box and one PBE-box, provides an interesting insight. Neither the competitor mutated in both motifs (Figure 1G; also Figure S2), nor the competitor mutated only in the G-box motif (Figure S3) displayed competitive activity, whereas the probe mutated only in the PBE-box motif showed competitive efficiency similar to that of the WT sequence (Figure S2). These findings suggest that PIF3 may have differential binding affinity toward these two motifs in specific genomic contexts.

### Identification of genes associated with PIF3-binding sites

Although all ChIP-defined transcription factor binding sites may prove to be functionally significant, we have chosen here to focus on identifying those genes displaying motif-coincident PIF3-binding sites located in conventional promoter regions (defined here as “PIF3-bound genes”). Initially, from the 1064 binding sites defined above, we identified 709 sites that are both intergenic and G- and/or PBE-box-coincident (Table S2). For these 709 sites, we defined PIF3-bound genes as having a binding site in the 5′ flanking DNA, within 5 kb of the transcription start site (TSS), in the absence of intervening genes. This analysis identified 596 PIF3-binding sites, with 828 associated genes, where some sites are associated with two genes on opposite strands. Of these genes, 88% have PIF3-binding sites within 3 kb of TSS, whereas the remaining 12% have sites between 3 and 5 kb upstream (Table S3). These 828 genes thus constitute a set of PIF3-bound genes whose transcription is potentially directly regulated by PIF3.

**Figure 2:**
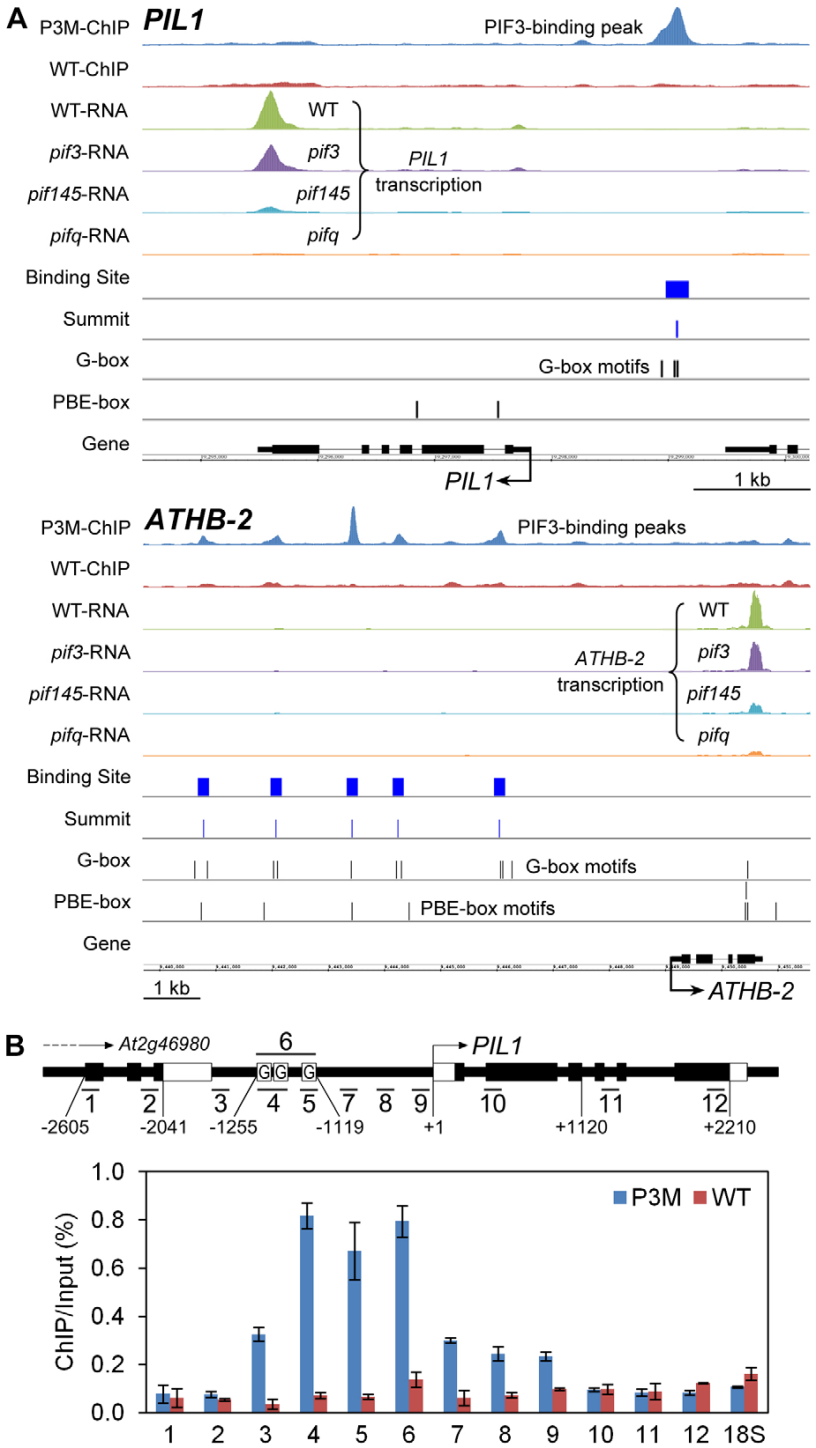
Compiled ChIP–seq and RNA–seq data identify *PIL1* and *ATHB2* as direct targets of PIF3 transcriptional regulation. (A) Visualization of ChIP-seq and RNA-seq data in the genomic regions encompassing two representative genes, *PIL1* and *ATHB-2*. The ChIP and RNA tracks show the pile-up distribution of the combined raw reads from four biological replicates of ChIP-seq data and three replicates of RNA-seq data, respectively. P3M- and WT-ChIP: DNA immunoprecipitated from PIF3-Myc-expressing and from wild-type seedlings, respectively. WT-, *pif3-*, *pif145-* and *pifq*-RNA: RNA from genotypes used for expression analysis. Binding Site: 201 bp defined as the PIF3-binding site. Summit: predicted PIF3-binding center defined from the binding-peak maximum. G- and PBE-box: Vertical lines indicate motif positions. (B) ChIP-qPCR verification of specific PIF3 binding to the G-box-located promoter region of *PIL1*. The schematic diagram illustrates the genomic region around the *PIL1* locus. The short bars with numbers show 12 specific qPCR products. The black and white rectangular boxes represent CDS and UTR, respectively. Boxes labeled ‘G’ indicate the approximate locations of three G-box motifs in the *PIL1* promoter. The relative enrichment level is represented by the percentage of co-immunoprecipitated DNA to the input control in the P3M and WT samples. Data are represented as the mean of biological triplicates +/− SEM. 18S: *18S rRNA* as internal control.

To provide genome-wide visualization of the ChIP-seq analysis, we developed a platform using the Integrated Genome Browser [39]. Figure 2A shows the chromosomal regions around *PIL1* and *ATHB-2*, as examples. The chromosomal region surrounding the *PIL1* gene shows a single PIF3-binding peak that is coincident with three G-box motifs located in the *PIL1* promoter region. *ATHB-2* is somewhat unusual in that it displays five specific PIF3-binding peaks in its extensive 5′-upstream region, each coincident with 1 to 3 G-box motifs (Figure 2A). ChIP-qPCR analysis scanning across the *PIL1* genomic region provides robust validation of the ChIP-seq data for this gene (Figure 2B).

### Definition of PIF3 contribution to the transcriptional pattern collectively regulated by the PIF-quartet during skotomorphogenic development

**Figure 3:**
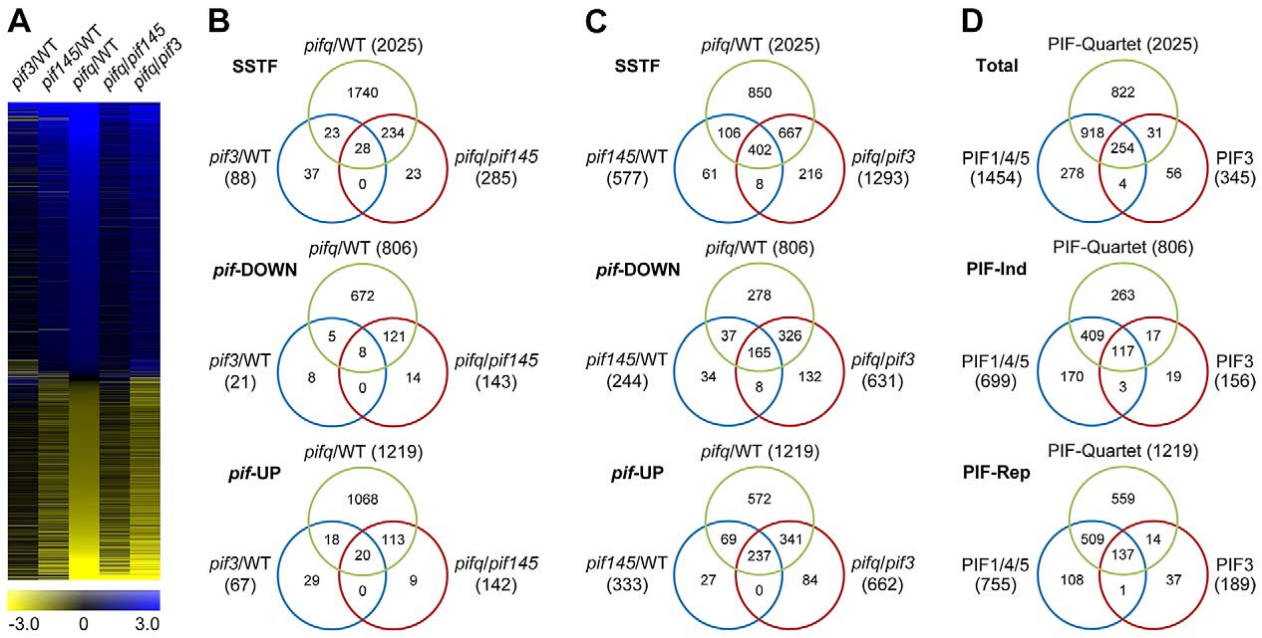
RNA–seq analysis of selected *pif*-mutants identifies PIF-regulated genes genome wide. (A) Hierarchical clustering of differentially expressed genes by fold-change in expression (log2 scale), in the five pairwise genotypic comparisons indicated (*pif3*/WT, *pif145*/WT. *pifq*/WT, *pifq*/*pif145* and *pifq*/*pif3*). Data shown are for the genes identified here as displaying Statistically-Significant, Two-Fold (SSTF) differences in any pairwise comparison. (B and C) Venn diagrams depicting total gene numbers (parentheses) and genes in common (overlapping sectors) that display SSTF differences in expression in the pairwise genotypic comparisons shown (Top Venn). Separation of genes into down-regulated (*pif*-DOWN; Middle Venn) and up-regulated (*pif*-UP; Bottom Venn) is based on the direction of the change in expression displayed in each pairwise comparison. (D) Venn diagrams summarizing the overlap between all identified PIF-quartet-, PIF1/4/5-trio- and PIF3-regulated genes. PIF3-regulated genes are defined as the combined total of the non-overlapping genes in the *pif3*/WT and *pifq*/*pif145* sets in (B). PIF1/4/5-trio-regulated genes are defined as the combined total of the non-overlapping genes in *pif145*/WT and *pifq*/*pif3* sets in (C). Separation of genes into PIF-induced (PIF-Ind; Middle Venn) and PIF-repressed (PIF-Rep; Bottom Venn) is based on the deduced action of the PIF proteins when present in WT seedlings.

To identify the genes regulated by PIF3, genome-wide, in the promotion of skotomorphogenic development, we performed 3′-end-capture directional RNA-seq analysis, comparing the expression profiles of 2-d dark-grown WT, *pif3*, *pif1pif4pif5* (*pif145*) and *pif1pif3pif4pif5* (*pifq*) *Arabidopsis* seedlings. Genes displaying Statistically-Significant Two-Fold (SSTF) expression changes in the three mutant genotypes compared to the WT and each other were identified as being regulated by the relevant mutated PIF(s) (Figure 3; listed in Tables S4, S5, S6, S7, S8). The degree of overlap between SSTF genes identified in these comparisons is depicted in the Venn diagrams in Figure 3B, 3C and 3D. We defined a combined total of 345 genes in the *pif3*/WT and *pifq*/*pif145* gene-sets as the composite PIF3-regulated gene-set (Table S9). Similarly, a combined total of 1454 genes in the *pif145*/WT and *pifq*/*pif3* gene-sets were defined as the composite PIF1/4/5-trio regulated gene-set (Table S10). Comparison of these composite gene-sets is displayed in Figure 3D.

The data indicate that 254 (74%) PIF3-regulated genes are also redundantly transcriptionally regulated by one or more of the other three PIF-quartet proteins. Conversely, 1740 (86%) PIF-quartet-regulated genes show no significant PIF3 dependence (Figure 3B), whereas 918 (45%) do display PIF1/4/5 regulation (Figure 3D), indicating that one or more of the other PIF-quartet members function non-redundantly with PIF3 in regulating the expression of many target genes. The general robustness of our genome-wide RNA-seq expression profiling is demonstrated by the extensive RT-qPCR validation data presented in Figure S3.

### Definition of genes that are likely direct targets of transcriptional regulation by PIF3

To identify the genes that both physically bind PIF3 in their promoters, in a G-box- or PBE-box-coincident manner, and display PIF-regulated expression (defined here as “direct-target genes”), we merged our ChIP-seq and RNA-seq data. This permitted gene-by-gene visualization of the PIF3-binding peaks and PIF-dependent transcription, genome-wide, as shown for *PIL1* and *ATHB2* in Figure 2A. The expression data for these two genes show a clear difference in transcript levels between the WT and *pifq* mutant, demonstrating the robust dependence of full expression on the presence of the PIF-quartet. Comparison of the expression peaks for the *pif145* and *pifq* mutants also suggests that PIF3 acts in the absence of the other three quartet members to promote a moderate increase in transcript levels. Overall, the combined PIF-regulated expression-patterns and promoter-located PIF3-binding sites displayed by these two genes render them likely direct-targets of transcriptional regulation by PIF3 and one or more other quartet members in promoting skotomorphogenic development.

**Figure 4:**
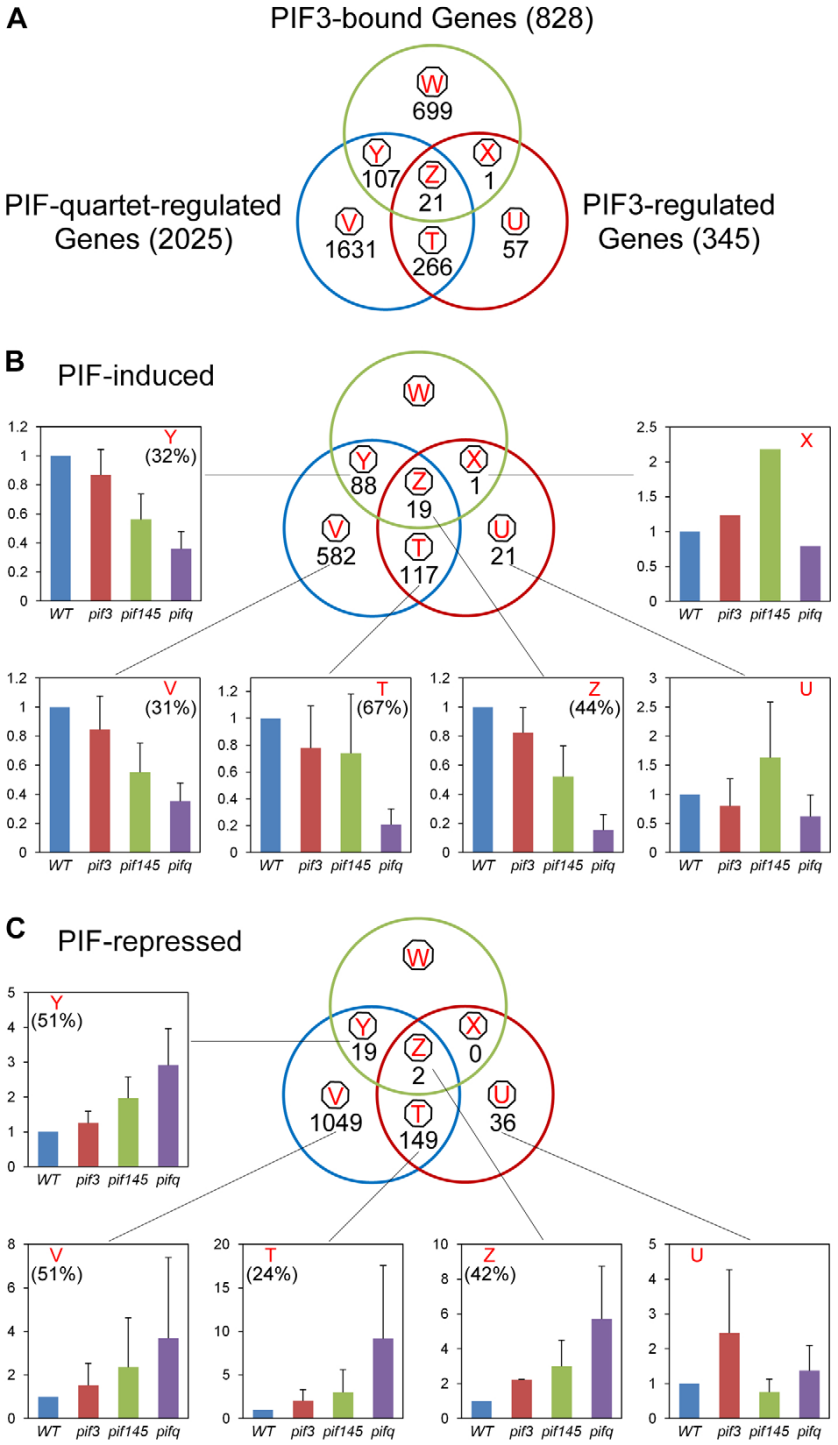
Merging of ChIP–seq and RNA–seq data identifies apparent direct targets of PIF3 transcriptional regulation. (A) Venn diagrams showing the overlap of genes displaying promoter-localized, G- or PBE-box-coincident, PIF3-binding peaks (PIF3-bound genes), with those displaying PIF-quartet- and/or PIF3-regulated expression (as defined in Figure 3D). This comparison defines seven classes of genes corresponding to the diagram sectors (circled red letters). The number of genes in each class is indicated. (B and C) Genes indicated in (A) divided into PIF-induced (B) and PIF-repressed (C) sets based on the direction of the transcriptional response elicited by the designated PIFs in dark-grown WT seedlings. The average fold-change in expression of all genes in each class relative to WT (set at unity) is shown in the bar graphs. Error bars represent the standard error for the genes averaged in each class. The percentage indicates the relative contribution of PIF3 to the total PIF-regulated expression in each class, defined by comparing *pif145* with *pifq*.

The Venn diagrams in Figure 4 show the genome-wide overlap of the genes identified independently as displaying PIF-quartet- and/or PIF3-dependent expression in a SSTF manner, with those exhibiting promoter-located, motif-coincident PIF3-binding sites (Figure 4A, Classes X, Y and Z; listed in Table S11). By these criteria, a total of 22 genes (Classes X and Z) were identified as robustly-likely, direct-target genes of autonomous-PIF3 transcriptional regulation. Of these, 21 genes (19 PIF3-induced; 2 PIF3-repressed) also display collective PIF-quartet regulation (Class Z). The 19 PIF3-induced Z-Class genes are listed in Table 1. The bar graphs in Figure 4B and 4C portray the mean expression level (relative to WT) of all the genes in each class, for each *pif* genotype. The quantitatively robust responsiveness of the PIF3-bound genes to the presence of PIF3 in the *pif145* mutant background is evident from these data (Class Z-associated bar graphs). This robust PIF3-responsiveness was validated using RT-qPCR for selected members of the 19 PIF3-induced, Z-Class gene-set, having a range of quantitative dependence on this bHLH factor (Figure S3A).

**Table 1:** Direct-target genes of PIF3-induced transcription (Class Z and YZ1.5 genes).

Functional Category/Locus	Annotation	Fold Induction (FI)				Transcriptional-response Category		
		PIF-quartet Induction	PIF3 Induction		PIF145 Induction	Direct PIF3 Induction^d^	Rapid Light Repression^e^	Reciprocal Light/Shade Regulation^f^
			PIF3 L.O.F.^a^	PIF3 G.O.F.^b^	PIF145 G.O.F.^c^			
		(WT/*pifq*)	(WT/*pif3*)	(*pif145*/*pifq*)	(*pif3*/*pifq*)			
**Transcription**								
AT2G46970	PIL1/bHLH124	97.58	1.52	17.71	65.55	Class Z	Not ATH1^g^	Class M
AT5G54470	BBX29	21.04	1.01	7.20	21.19	Class Z		
AT1G02340	HFR1/bHLH26	24.56	1.39	4.41	18.00	Class Z		
AT3G21330	bHLH87	3.09	1.17	2.07	2.69	Class Z	Not ATH1	
AT4G16780	ATHB-2	7.36	1.25	2.06	5.98	Class YZ1.5	Class 7	Class M
AT3G61830	ARF18	3.59	1.33	2.03	2.75	Class YZ1.5	Class 7	
AT4G27310	BBX28	2.93	1.28	2.00	2.33	Class YZ1.5	Not ATH1	
AT5G53980	ATHB52	3.72	1.20	1.92	3.15	Class YZ1.5	Class 7	Class M
AT4G32280	IAA29	6.43	1.03	1.91	6.35	Class YZ1.5	Class 7	Class M
AT4G01250	WRKY22	2.62	1.18	1.80	2.25	Class YZ1.5		
AT2G42870	PAR1/HLH1	2.01	0.96	1.69	2.13	Class YZ1.5		
AT1G69690	TCP15	2.53	1.06	1.66	2.43	Class YZ1.5	Class 7	Class M
AT3G25730	EDF3	4.01	1.61	1.66	2.53	Class YZ1.5	Class 7	
AT3G15540	IAA19/MSG2	3.21	1.15	1.66	2.84	Class YZ1.5	Class 7	Class M
AT1G77200	AP2-EREBP family	2.96	1.28	1.66	2.36	Class YZ1.5	Class 7	
AT3G62090	PIF6/PIL2/bHLH132	13.38	1.15	1.51	11.79	Class YZ1.5	Class 7	
**Stress/Defense**								
AT2G33380	RD20/CLO3	25.22	1.96	15.47	13.07	Class Z		
AT5G63650	SNRK2.5	6.72	1.23	3.62	5.53	Class Z	Class 7	Class M
AT4G36010	Thaumatin family	3.74	1.30	1.83	2.91	Class YZ1.5	Class 7	Class M
**Signaling**								
AT3G48260	WNK3	8.54	1.28	3.33	6.78	Class Z	Not ATH1	
**Photosynthesis**								
AT1G34630	Tim17/22/23 family	3.31	1.52	1.87	2.22	Class YZ1.5	Class 7	
**Growth/Development**								
AT4G14130	XTR7/XTH15	19.37	1.00	6.07	19.66	Class Z	Class 7	Class M
AT1G67265	RTFL21/DVL3	9.92	1.21	2.71	8.31	Class Z	Not ATH1	
AT1G10550	XTH33	3.11	1.46	2.21	2.16	Class Z	Class 7	
**Cellular Metabolism**								
AT5G04120	dPGM-like	9.35	1.53	8.04	6.22	Class Z		
AT4G11050	GH9C3	11.38	1.40	6.90	8.25	Class Z	Class 7	
AT5G51210	OLEO3	6.54	1.54	4.42	4.32	Class Z		
AT2G31980	CYS2	7.85	0.80	3.28	9.92	Class Z	Class 7	
AT5G02540	SDR	12.09	1.40	2.18	8.76	Class YZ1.5	Class 7	Class M
AT2G34020	Ca-binding EF-hand	2.14	1.04	1.96	2.10	Class YZ1.5	Class 7	
AT5G07010	ST2A	4.80	0.92	1.95	5.32	Class YZ1.5	Class 7	
**Unknown**								
AT4G35720	Unknown protein	8.30	1.04	3.98	8.10	Class Z	Class 7	Class M
AT5G02580	Unknown protein	4.68	1.01	2.38	4.68	Class Z	Class 7	Class M
AT3G17580	Unknown protein	3.94	1.05	2.17	3.82	Class Z	Not ATH1	
**Non-coding**								
AT5G26146	Natural antisense	3.08	1.35	2.63	2.33	Class Z	Not ATH1	
AT3G17110	Pseudogene	2.97	1.10	2.35	2.74	Class Z		

a PIF3 loss-of-function (L.O.F.);

b PIF3 gain-of function (G.O.F.);

c PIF1/4/5-trio gain-of function (G.O.F.);

d See Figure 4 and Figure S3;

e See [17];

f See [11];

g Gene not represented on the Affymetrix ATH1 array.

A striking feature of our data is the relatively large number of PIF-quartet-regulated genes (107 total genes; 88 PIF-induced and 19 PIF-repressed) that display promoter-located, PIF3-binding sites, but lack evidence of SSTF-level PIF3 regulation in our RNA-seq analysis (Figure 4, Class Y; also Table S11). Nevertheless, the bar graphs of the mean expression of these genes suggest a tendency toward a consistent difference in expression between the *pif145* and *pifq* mutants, across the gene-set. In addition, combined analysis of the full set of PIF-induced, PIF3-bound genes (Classes Y and Z together) shows that there is a reciprocal continuum in the magnitude of the relative contributions of PIF3 and the PIF1/4/5-trio to the collective activity of the PIF quartet in transcriptionally activating these genes (Figure S4). To more closely examine the PIF3 contribution to the total PIF-quartet activity in the Y-Class genes, we therefore arrayed these genes by the *pif145*/*pifq* fold-change value and assayed the relative expression levels in 20 selected PIF-induced loci by RT-qPCR. Figure S3B shows that the 17 genes in this group with fold-changes >1.5 by the RNA-seq analysis, all exhibit statistically-significant (Student's *t*-test, *P*<0.05), PIF3-promoted expression increases in the *pif145* mutant compared to the *pifq* mutant by RT-qPCR. This suggests that a subset of Y-Class genes may represent additional *bona fide* autonomously-PIF3-regulated genes that are below the resolution of the SSTF criteria we imposed on our RNA-seq analysis. We have therefore designated these 17 as Class YZ1.5 genes, having moderate (>1.5-fold), but statistically significant, regulation by PIF3 (Table 1). The evidence indicates, therefore, that a combined total of at least 38 YZ1.5- and Z-Class genes are direct targets of moderate to robust transcriptional regulation by promoter-bound PIF3. Because an additional 34 of the 88 Y-Class genes also display >1.5-fold PIF3-induced expression (Figure S3B and Table S11), it is possible that the number of direct targets of partial PIF3 transcriptional regulation is yet larger.

The W-class genes are those that display promoter-localized, G- or PBE-box-coincident PIF3-binding peaks, but no differential expression between the *pifq* mutant and wild type (Figure 4A). This observation is consistent with data from a variety of organisms that have shown that transcription factors vary greatly in their number of genomic binding sites, and that binding events can vastly exceed the number of known or possible direct gene targets [40]. The reasons for this phenomenon here are unclear but could include functional redundancy with other factors, including other PIF proteins. Consistent with this possibility, a subset of 41 of the total 699 W-class genes exhibit rapid light responsiveness [17] upon initial exposure (Table S12).

### Definition of genes that are potential direct targets of transcriptional regulation by PIF1, 4, and/or 5, as well as PIF3

The reciprocal continuum in relative PIF3 and PIF1/4/5-trio contributions to the collective PIF-quartet transcriptional activation of PIF-induced, Y- and Z-class genes referred to above (Figure S4), indicates that PIF1, 4 and/or 5 contribute substantially to the regulation of these PIF3-bound genes. To identify the individual genes in this set displaying a significant PIF1/4/5 contribution, we compared the Y- and Z-class genes (Table S11) with those defined above as PIF1/4/5-regulated (Figure 3D; also Table S10). Overall, 92 (72%) of the 128 combined Y- and Z-class genes exhibit regulation by PIF1, 4 and/or 5 (Table S11), as shown by significant differences in the *pif145*/WT and/or *pifq*/*pif3* comparisons. More notably, all 38 PIF3-induced direct-target genes (Class YZ1.5 and Z) are also PIF1/4/5-induced (Table 1). Because all four PIFs have been shown to bind to the G-box motif in sequence-specific fashion [4]–[9], it appears probable that these PIF-quartet-regulated genes, displaying promoter-located PIF3-binding sites (Figure 4; also Table S11), may be directly regulated by one or more of the other quartet members, in addition to, or instead of, PIF3.

### Transcription-factor genes are the dominant targets of direct transcriptional regulation by the PIFs

Categorization of the YZ-class genes by the known or predicted functions of their encoded products reveals substantial enrichment in a diversity of transcription-factor-encoding genes (Table 1; also Figure S5 and Table S11), consistent with the concept that these multiple direct targets of the PIF quartet function at the apex of a primary transcriptional-cascade to regulate the downstream transcriptional network. It is also notable, however, that a considerable number of the YZ-class genes that have other cellular functions are also apparent direct targets of transcriptional regulation by the PIFs, including two non-protein-encoding genes of unknown function (Table 1).

### Definition of light-regulated direct-target genes of PIF3

Previously, by microarray profiling, we identified a subset of genes (designated Class 7) that, in dark-grown seedlings, exhibit a PIF-quartet-dependent expression pattern, that is rapidly reversed (within 1 h) upon initial exposure to phy-activating R light [17]. Of the 24 rapidly light-repressed Class 7 genes displaying promoter-localized, G- or PBE-box-coincident PIF3-binding peaks, 21 (88%) are either Class YZ1.5 or Z genes here (Table 1). These genes are thus identified as a subset whose expression is directly promoted, at least partially, by PIF3 transcriptional activation in the dark, and is rapidly reduced in the light, at least in part, by photoactivated-phyB-induced PIF3 degradation. It is notable that 9 of these genes (43%) encode transcription factors (Table 1), indicative of being master regulators at the apex of the downstream transcriptional cascade controlled by the phy signaling pathway.

In striking contrast to the light-repressed Class 7 genes, only 7 of the 115 rapidly light-induced Class 7 genes (6%) [17] display PIF3-binding peaks that are coincident with a G-box or PBE-box, and of these only 2 genes (<2%) (*PSY* and *KAI2*) exhibit derepression here in the dark-grown *pifq* mutant. No individual PIF3 contribution to this repression was detectable here. Collectively, these data indicate that PIF3 acts predominantly, if not exclusively, to activate the expression of direct-target genes in dark-grown seedlings. Conversely, the 94% (108/115) of light-induced Class 7 genes that do not display G- or PBE-box coincident PIF3-binding peaks, might suggest that one or more of the proposed direct targets of the PIF quartet (Table 1) can act as key repressor(s) that regulate a diverse set of light-induced genes.

Recently, we defined a small core set of 14 Class 7 PIF-quartet-regulated genes (called M-Class genes) that display rapid, reciprocal, transcriptional responsiveness to light and vegetative shade in dark-grown and light-grown seedlings, respectively [11]. Our present analysis shows that 11 (79%) of these M-Class genes are identical to those identified here as dual Class 7 and YZ1.5-/Z-Class genes (Table 1), indicating that they are likely direct targets of PIF3 regulation, not only during skotomorphogenesis and deetiolation, but also subsequently, on a continuing basis, through juvenile vegetative development. The correlated PIF3-binding and PIF-regulated transcriptional behavior of several of these M-Class genes, determined by merging the ChIP-seq and RNA-seq data, is depicted in Figure S6.

### Individual PIF-quartet members display quantitatively differential regulation of different direct-target genes

**Figure 5:**
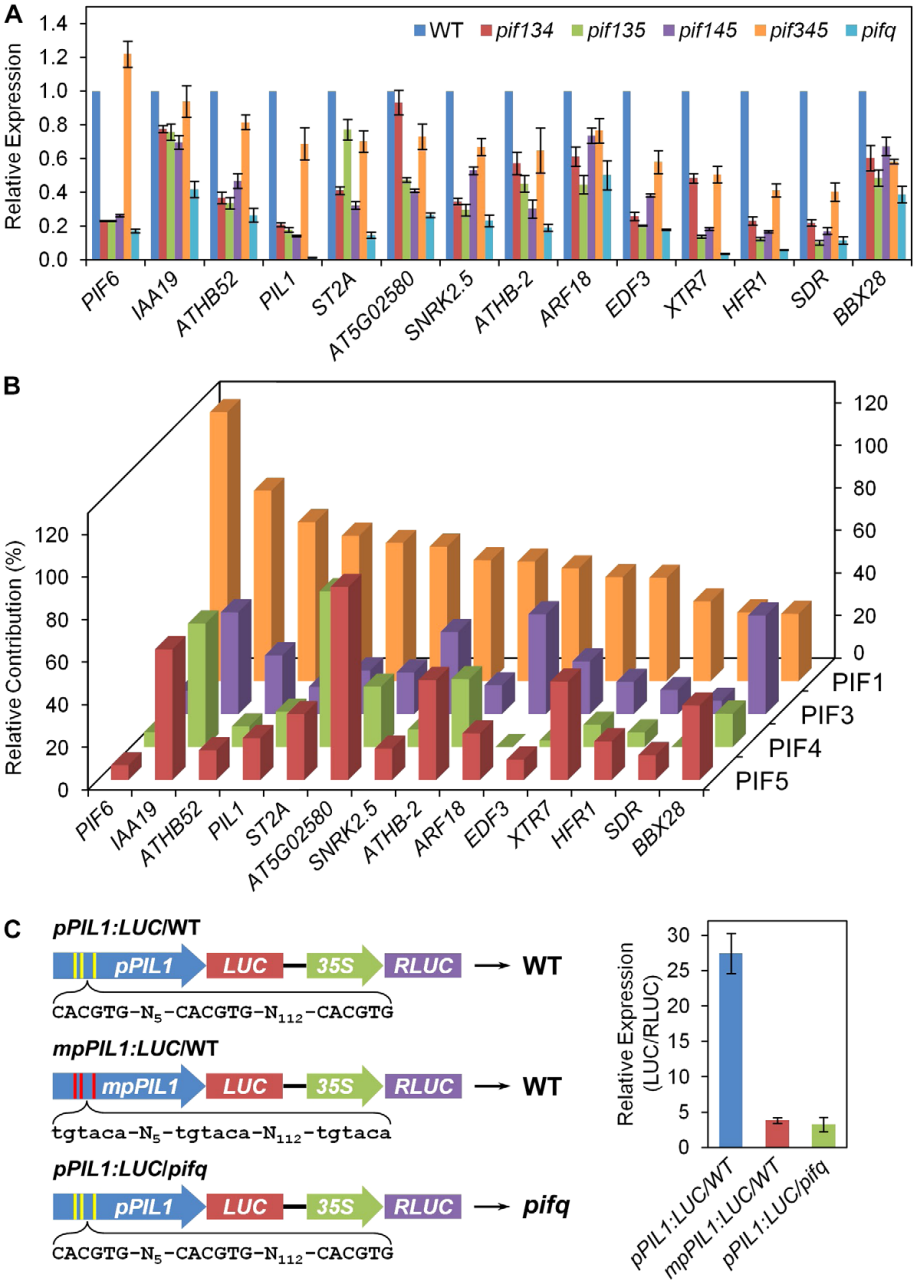
Differential regulation of PIF3 direct-target genes by individual PIF-quartet proteins. (A) Individual PIF-quartet members display diverse patterns of shared regulatory activity toward genes defined as direct targets of PIF3 transcriptional activation. Expression levels in the *pifq* and *pif*-triple mutants indicated, were determined by RT-qPCR, normalized to an internal *PP2AA3* control, and presented relative to WT levels set at unity. Data are represented as the mean of biological triplicates +/− SEM. (B) Matrix of relative contributions from individual PIF proteins toward the shared transcriptional activation of individual, potentially-shared direct-target genes. Percent contribution is calculated as the proportion of the total differential expression between *pifq* and WT, that is contributed by the differential expression between *pifq* and each *pif*-triple mutant. (C) *In planta PIL1* promoter activity requires both G-box motifs and PIF-quartet members. Left: Schematic of *pPIL1:LUC* constructs expressed transgenically in either WT or *pifq* plants, as indicated. Yellow and red stripes represent the locations of three native (*pPIL1*) and mutated (*mpPIL1*) G-box motifs, respectively, in variants of the *PIL1* promoter, as shown by the DNA sequences displayed below each construct. A contiguous 35S-promoter driven *RLUC* reporter was included as an internal control in each construct. Right: Mean expression of the *LUC* reporter gene is shown as LUC enzyme activity normalized to the RLUC control in the same transgenic plant. Data represent the means of 6 or 7 independent transgenic lines +/− SEM.

Because our data indicate that the contribution of PIF3 to the total level of expression collectively regulated by the PIF-quartet is quantitatively variable between genes (Figure 2A; Figure 4B, 4C; Figures S3 and S4), we wished to determine whether the other members of the PIF-quartet display a similarly variable pattern of regulation. For this purpose, we assayed the expression by RT-qPCR of a selected set of apparently PIF3 direct-target genes (Classes Z and YZ1.5), in the four different *pif* triple mutants compared to the *pifq* mutant and WT (Figure 5A). The relative autonomous contribution of each individual PIF (in the absence of the other three quartet members) to the total, collective PIF-quartet-supported expression was calculated as a percentage of the total difference in expression between the WT and *pifq* mutant, for each separate gene. The data reveal a striking diversity of relative contributions, both between the individual PIFs, and between genes for any individual PIF, in two-dimensional-matrix fashion (Figure 5B). Particularly notable is the dominant role played by PIF1 in promoting the expression of the majority of these genes. On the other hand, PIF3 contributes strongly to *ARF18*, *SNRK2.5* and *BBX28* expression, PIF4 strongly activates *ST2A* and *ATHB-2*, PIF5 contributes actively to *AT5G02580*, *ATHB-2* and *XTR7* expression, while all four PIFs contribute substantially to *IAA19* transcription. Because all these tested genes are prospective direct-target genes of multiple PIF-quartet members, our findings suggest that there is an intricate combinatorial network, in which the individual PIF-quartet factors collaborate to transcriptionally regulate an array of direct-target genes, through potentially common DNA binding sites, with quantitatively differential regulatory activity.

Comparison of our data with recently published ChIP-seq-identified PIF4- and PIF5-binding sites [21], [24] supports this conclusion (Figure S7). Although the three studies were performed under contrasting experimental conditions, our analysis shows that 82% of genes with promoter-located, G- or PBE-box-associated PIF3-binding peaks identified here, also display PIF4- and/or PIF5-binding peaks (Figure S7A). Perhaps more striking, 89% of the 128 PIF3-binding, PIF-quartet-regulated genes identified here (Y- and Z-class genes in Figure 4A), are also bound by PIF4 and or PIF5, with 52% being bound by all three PIFs (Figure S7B; Table S11).

**Figure 6:**
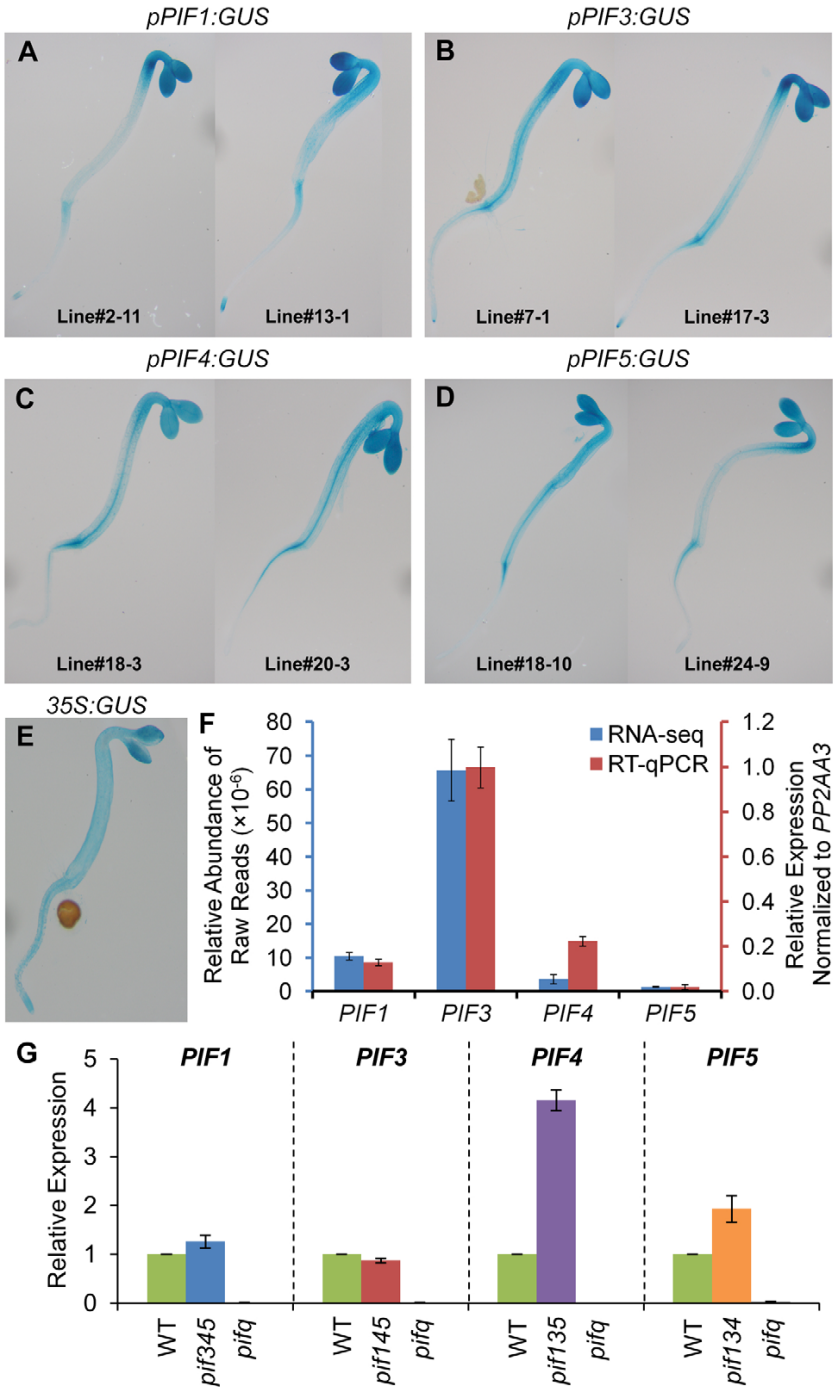
Expression patterns of PIF-encoding genes. (A–E) Representative images of histochemical staining of GUS activity in 2-d-old dark-grown transgenic seedlings. The *GUS* reporter gene is driven by *PIF1* (A), *PIF3* (B), *PIF4* (C), *PIF5* (D) and *CaMV 35S* (E) promoters, respectively. Data of biological triplicates were collected from two independent transgenic lines, and representative images are shown for each transgene. (F) Steady-state transcript levels of PIF-encoding genes defined by RNA-seq and RT-qPCR analyses in 2-d-old dark-grown WT seedlings. Data are presented as the mean of biological triplicates +/− SEM. (G) Relative expression of PIF-encoding genes in 2-d-old dark-grown *pifq* and *pif*-triple mutants. Expression was determined by RT-qPCR, normalized to an internal *PP2AA3* control, and presented relative to WT levels set at unity. Data are represented as the mean of biological triplicates +/− SEM.

One possible mechanistic basis for the differential control of shared target genes by the individual PIF-quartet members described above (Figure 5) is that each PIF transcription factor has a different spatial expression pattern across the plant. To examine this possibility, we expressed *pPIF:GUS* fusions for each of the *PIF* genes transgenically in *Arabidopsis* seedlings, and assayed the distribution of GUS expression histochemically. The data show that all four *PIF* promoters support expression broadly throughout the seedling shoot tissue, with largely similar distribution patterns between the quartet members, within the resolution of this procedure (Figure 6A–6D).

In principle, differences in absolute expression levels among the PIF-quartet members could also be a fundamental determinant of differences in PIF-promoted expression of target genes (Figure 5). However, this does not appear to be the case here. Examination of the RNA-seq profiles, and independent RT-qPCR analyses, of *PIF1*, *PIF3*, *PIF4* and *PIF5* expression, shows that, while there are marked differences in expression between these genes in wild-type seedlings (Figure 6F), these are not strongly correlated with the respective patterns of target-gene expression (Figure 5). In particular, *PIF1* and *PIF3* display expression levels that are robustly converse to their respective general levels of transcriptional activation. Similarly, and more importantly, although the expression levels of *PIF4* and *PIF5* are significantly elevated in the relevant triple mutant compared to wild-type (Figure 6G), these differences also do not correlate with the overall differential expression patterns of the target genes. While these elevated levels could indicate that the computation in Figure 5B overestimates the normal, relative contributions of these two PIFs to the collective PIF-quartet activity, displayed when all four PIFs are present, they do not account for the apparent dominance of PIF1 or the diversity of response-patterns between the genes.

Taken together, these results suggest that the sometimes strikingly different quantitative contributions of the individual PIFs to the expression of a given target gene appears unlikely to be primarily due to either differences in transcriptionally-driven PIF abundance or differences in spatially-determined abundance of the PIF-quartet-members. It appears more likely that these differences are due to intrinsic differential activities of the individual PIFs in the context of the individual target-gene promoters. In addition, because the *GUS* expression pattern driven by the CaMV *35S* promoter (Figure 6E) overlaps substantially with that driven by the *PIF* promoters (Figure 6A–6D), it seems reasonable to expect that the majority of PIF3-binding sites detected by ChIP-seq analysis here, using *35S*-driven PIF3-Myc expression, will reflect sites that are normally available to PIF3 generated by endogenous *PIF3* promoter activity.

### The G-box motifs in the *PIL1* promoter are functionally necessary for PIF-quartet promoted expression

The robust binding of PIF3 to the G-box-containing region of the *PIL1* promoter detected by ChIP-seq analysis (Figure 2) and *in vitro* assay (Figure 1G and Figure S2), and the partial autonomous promotion of *PIL1* expression by PIF3 observed by RNA-seq analysis (Figure 2A, Figure 5, and Figure S3A), provides strong evidence that *PIL1* is a direct target of PIF3 transcriptional regulation via physical interaction of the bHLH factor with these *cis*-elements. Conversely, because the non-PIF3 quartet members contribute robustly to the collective PIF-quartet-dependent expression of *PIL1* (Figure 2A and Figure 5), and given that these non-PIF3 members also bind selectively to G-box motifs [4]–[9], it might be predicted that PIF1, 4 and/or 5 transcriptional activation of *PIL1* will, like PIF3, be exerted through interaction with the G-boxes in the *PIL1* promoter [5], [6]. To examine this prediction, we tested the functional necessity of these G-box motifs to *PIL1* expression using reporter constructs in transgenic seedlings. The data show that activation of the *PIL1* promoter requires both the presence of one or more of the PIF quartet and one or more of the G-boxes (Figure 5C), indicating that the G-box elements are the major, if not sole, targets of PIF-quartet transcriptional activation activity. By contrast, it is notable that, although a recent report shows that PIF7 also binds to the G-box region of the *PIL1* promoter in a manner that is functionally important for shade-induced expression of this gene in light-grown seedlings [41], the extremely low residual levels of *PIL1* expression in dark-grown *pifq* seedlings compared to wild-type (Figure 2A and Figure 5A) indicate that PIF7 has minimal, if any, contribution under these conditions. Together, the evidence suggests that, to the extent that the PIF-quartet members share transcriptional activation of *PIL1* (Figure 5A and 5B), they do so by sharing the G-box motifs as interaction sites. This conclusion is consistent with the demonstration that PIF3 binds to all three G-box motifs in the *PIL1*-promoter cluster, both *in vivo* (Figure 2B) and *in vitro* (Figure 1G and Figure S2). By extrapolation, the other Y- and Z-Class, PIF-quartet-regulated genes, established here as being direct-targets of PIF3 transcriptional regulation through G- or PBE-box binding motifs, are strong candidates for likewise being targets of functionally active, direct binding-site sharing among the four PIF factors.

## Discussion

Previous genetic studies have established that the overarching biological function of the PIF quartet is to promote skotomorphogenic growth and development in post-germinative seedlings in darkness, and to promote shade-avoidance behavior in deetiolated seedlings in response to exposure to neighboring vegetation [2], [3], [11], [42]. The evidence shows that the quadruple *pifq* mutant is strongly impaired in skotomorphogenic growth and development in dark-grown seedlings and has reduced shade-avoidance responsiveness to signals from neighboring vegetation in green seedlings [2], [3], [11]. In addition, there are indications that the contributions of individual PIF members to the collective activities of the quartet vary quantitatively, both between the PIFs for a given morphogenic-response feature, and between morphogenic-response features for a given PIF. For example, experiments comparing single, double, triple and quadruple *pif*-mutant combinations indicate that the individual PIFs appear to contribute additively or synergistically, in more or less equivalent fashion, to the promotion of hypocotyl-cell elongation growth in dark-grown seedlings [2], [3], [11]. By contrast, PIF1 appears to dominate the concomitant suppression of cotyledon separation that occurs in these same seedlings during dark-growth [2], [11]. In green seedlings, on the other hand, PIF4 and/or PIF5 appear to have a major role in promoting the stem and petiole elongation intrinsic to shade-avoidance in response to vegetative shade [21], [42], whereas PIF3 [43], together with PIFs 4 and 5 [44], contribute strongly to growth during the night period under diurnal light/dark cycles. Consistent with this general pattern, another related bHLH factor, PIF7, displays only moderate involvement in seedling deetiolation [45], but has a prominent role in shade avoidance [41]. Although a limited number of previous studies have examined the transcriptome regulated by PIF-quartet members in seedlings in darkness [3], [12], [16]–[19] and vegetative shade [11], [21] using the Affymetrix ATH1 array, these studies did not provide full genome coverage and did not permit dissection of potential quantitative differences in transcriptome profiles controlled by the individual PIFs.

The RNA-seq analysis performed here defines, with full genome coverage, the transcriptome collectively regulated by the PIF quartet in promoting skotomorphogenesis, and provides initial definition of the extent, and quantitative partitioning, of shared transcriptional control of the genes within of this network between PIF3 and the PIF1/4/5-trio. Superimposed on these data, our ChIP-seq analysis has identified a subset of these genes that are likely direct targets of PIF3 transcriptional regulation, exerted by physical binding of this factor to promoter-localized G- or PBE-box recognition-motifs (Class X, Y and Z genes, combined; Figure 4). The predominant pattern of PIF-regulated expression of these PIF3-bound genes (108 (84%) of 129 total) is one of high levels in the presence of the wild-type PIF factors, and reduced levels in the genetically-imposed absence of these factors in dark-grown seedlings, indicative of transcriptional activation by PIF3 and/or one or more of the other three PIF-quartet members. This pattern is consistent with the existing reports that all four factors function intrinsically as transcriptional activators, at least in transfection or heterologous expression systems [4]–[9], and with the demonstration here and elsewhere [21] that these PIFs function to activate *PIL1*-promoter-driven expression in transgenic seedlings (Figure 5C). We have therefore focused here primarily on this predominant class of PIF-transcriptionally-activated genes.

Our data indicate that there is a continuum, from robust to marginal, in the extent of the contribution of PIF3 to the combined transcriptional regulatory activity of the PIF quartet toward the PIF-induced, Class Y and Z genes (Figure S4). Conversely, by definition, there is a complimentary continuum in the share of this combined activity provided by the collective actions of PIFs 1, 4 and 5. These data imply at least some degree of shared, but quantitatively differential, transcriptional-regulatory activity among the PIF-quartet members toward individual genes that are apparent direct targets of PIF3-induced expression. Our RT-qPCR analysis of the expression patterns of selected genes from this subset, in all *pif* triple-mutant combinations, confirms that all four quartet members display such intra-subfamily differential activity toward individual genes in this set. Moreover, this analysis shows, conversely, that the individual PIF proteins induce differential levels of transcription in each different gene (Figure 5A). The three-dimensional response surface generated by this comparison (Figure 5B) suggests that this pattern may be iterated across all PIF-regulated genes genome wide, and points to the potential for considerable signaling and regulatory complexity at the PIF-target-gene interface.

Because it has been shown that all four PIF-quartet members bind robustly to the G-box motif [4]–[9], it appears likely that many of the direct targets of PIF3 transcriptional regulation are also direct targets of these other PIFs [5], [6], and that the shared activation of genes by the individual quartet members observed here will involve some degree of shared occupancy of these binding sites by the different PIFs. This may also apply to the newly discovered PBE-box motif, as there is recent evidence that PIF4 also recognizes this motif [21]. However, there is also evidence of potential divergence in motif recognition, as PIF5 was shown in the same report not to bind to the PBE-box motif [21]. The prominent contribution of PIF1 to the transcriptional activation of many of the genes examined here (Figure 5), despite its apparent considerably lower expression level than PIF3 (Figure 6F), is particularly intriguing in this respect, as this may imply that PIF1 may dominate promotion of target gene expression in dark-grown seedlings.

Comparison of the genes identified here as direct targets of PIF3 transcriptional activation (Class Z and YZ1.5 genes), with those previously identified as being rapidly (within 1 h) repressed by initial exposure of dark-grown seedlings to red light [17], has defined an overlapping subset of 21 genes (22 including *PIL1*) (Table 1). The evidence is strong, therefore, that these 22 genes form a core set that are directly transcriptionally activated by PIF3 in darkness and repressed in light, at least in part, by direct, photoactivated-phy-induced PIF3 degradation. Moreover, because all of these genes are also transcriptionally activated, either collectively (Table 1 and Table S11), or individually (Figure 5A and 5B) by PIF1, 4 and/or 5 in darkness, it appears likely that these PIFs share similarly directly in the light-reversible trans-activation of this core gene-set via photoactivated-phyB-induced degradation of the PIF-trio members.

The predicted or established functional diversity of the PIF direct-target genes identified here (Figure S5) suggests that PIF3 and/or one or more other PIF-quartet members act pleiotropically to directly regulate the transcription of a diversity of genes involved in a spectrum of cellular processes that sustain the skotomorphogenic developmental pathway. Consistent with previous analyses [3], [11], [17], [21], the PIF-induced genes are strikingly enriched for transcription-factor-encoding loci (40% of the annotated genes in this set). These data support the proposition, therefore, that the PIFs regulate an extensive transcriptional network via direct activation of a battery of primary target-genes in a hierarchal transcriptional cascade [20]. Because the encoded target-proteins represent multiple major classes of transcription factors (including bHLH, homeobox, bZIP, ARF, AUX/IAA, AP2-EREBP, BBX and TCP), it appears likely that they act concomitantly to activate multiple, diverse downstream pathways in parallel. Interestingly, however, many apparent PIF direct-target genes are involved in other cellular processes (including cytokinin metabolism, auxin-responsiveness, protein phosphorylation and cell-wall metabolism), suggesting a more immediate mode of PIF regulation of these processes.

**Figure 7:**
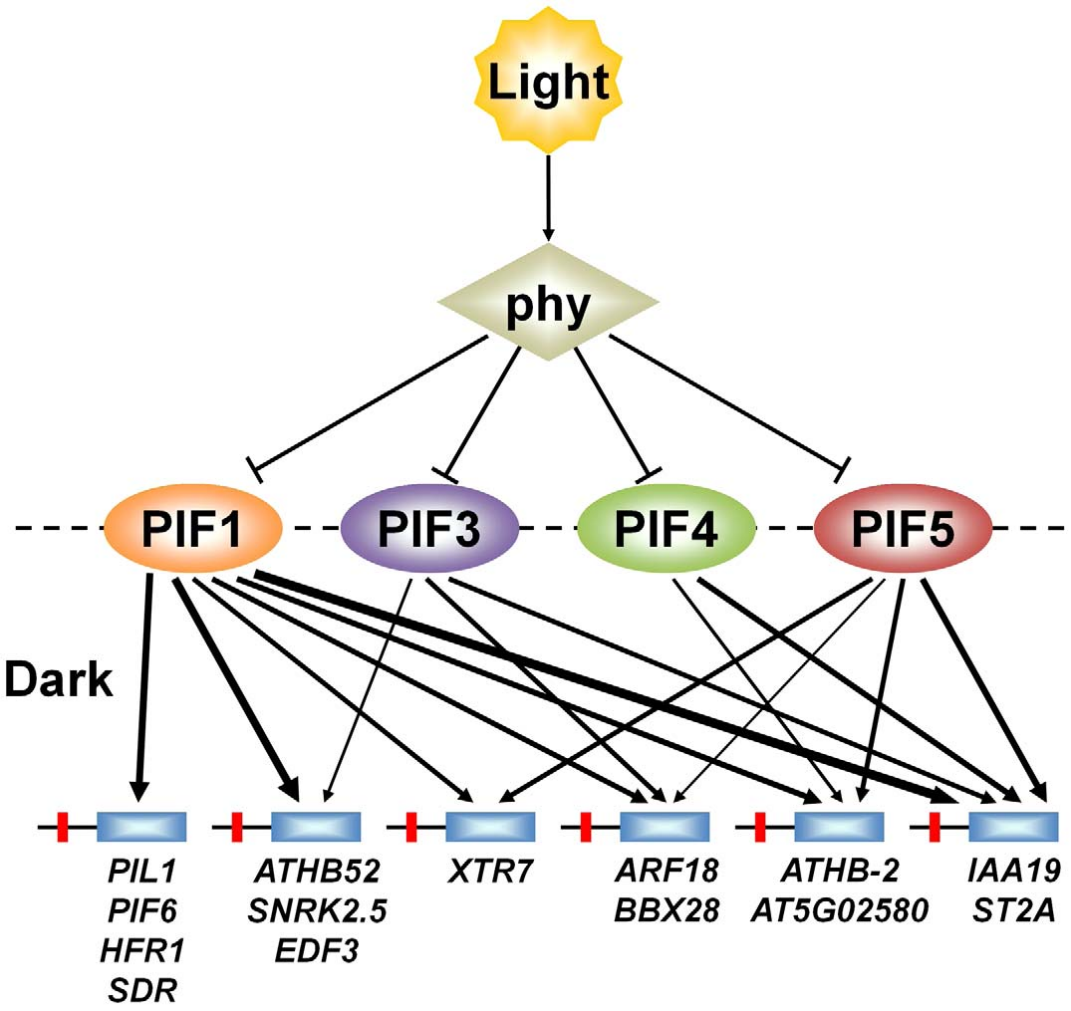
PIFs direct differential light-signal channeling to the phy-regulated transcriptional network. Model depicting proposed quantitatively differential partitioning of transcriptional activation activity to shared direct-target genes, both by and between individual PIF-quartet members. Arrows represent the presence or absence and relative level (line thickness) of shared transcriptional activation of different direct-target genes by the individual PIFs in the dark. This representation is based on the data in Figure 5A and 5B. Light-activation of phy photoreceptors induces rapid proteolysis of the PIFs, reversing this transcriptional activity.

A central issue in understanding mechanisms of eukaryotic transcriptional regulation is how members of large transcription-factor families, with conserved DNA-binding domains (such as the 162-member *Arabidopsis* bHLH family [46]), discriminate between target genes [22], [30], [47]. However, the specific question of whether, and to what extent, closely-related sub-family members, with potential overlapping functional redundancy (like the PIF quartet), share regulation of target genes through shared binding to promoter-localized consensus motifs, does not appear to have been widely investigated [31]–[34]. Our data, together with those of others [21], [24], provide evidence suggesting that the PIF quartet members share directly in transcriptional activation of numerous target genes, potentially via redundant promoter occupancy, in a manner that varies quantitatively from gene to gene (Figure 7). This finding suggests that these PIFs function collectively as a signaling hub, selectively partitioning common upstream signals from light-activated phys at the transcriptional-network interface. Definition of the mechanistic basis and functional consequences of this apparent complexity will require further investigation.

## Materials and Methods

### Plant materials and growth conditions

The Colombia-0 ecotype of *Arabidopsis thaliana* was used for all experiments. The *35S:6×His-PIF3-5×MYC* (P3M) transgenic line [48], *pif3* [18], *pif1pif4pif5* (*pif145*) [11], *pif1pif3pif4* (*pif134*), *pif1pif3pif5* (*pif135*), *pif3pif4pif5* (*pif345*), and *pif1pif3pif4pif5* (*pifq*) [2] were as described.

Stratified seeds were irradiated with WL at 21°C for 3 h to induce germination, followed by a FR pulse for 15 min to suppress pseudo dark effects [2], and grown in darkness at 21°C for 2 d before harvest.

### ChIP–seq

The ChIP assay was performed using about 2 g of *Arabidopsis* 2-d-old dark-grown whole seedlings as described [49] under green safelight. Polyclonal anti-MYC antibodies (Abcam, ab9132) were used with BSA-blocked Protein G Agarose beads (Millipore) to immunoprecipitate the P3M-DNA complex. Wild-type *Arabidopsis* seedlings grown under the same conditions were used as the negative control following the same assay procedure.

The ChIP-seq library was constructed according to Illumina's instructions (www.illumina.com) with some modifications. Four ChIP samples from technical replicates of each biological replicate were pooled together and concentrated to increase the starting amount of DNA. The end repair of DNA fragments was performed using End-It DNA End-Repair Kit (Epicentre). The A-tailing was added to the end-repaired DNA fragments using Klenow Fragment (NEB), and then Illumina's PE adapters were ligated by T4 DNA Ligase (Promega) at 16°C overnight. The adapter-ligated DNA fragments in the 200–300 bp size-range were selected by the gel purification, and then were amplified using Phusion High-Fidelity DNA Polymerase (NEB) with the Illumina PE PCR primer set. The library was purified using an Agencourt AMPure XP system (Beckman Coulter Genomics), and then validated by Bioanalyzer 2000 (Agilent).

The parallel libraries from P3M and WT ChIP samples were assayed by single-end sequencing on an Illumina GAIIx platform. The 36-nt reads were aligned to the TAIR9 assembly of the *Arabidopsis* genome using Bowtie [50] with up to 2 mismatches allowed. Only reads mapped uniquely to the nuclear genome with the lowest number of mismatches were retained for binding-peak identification. To increase the uniformity of read-counts across biological replicates, two technical-replicate sequencing runs were performed on the 4 libraries from the 1st and 2nd ChIP experiments (two of the four biological replicates). The aligned reads from the two technical sequencing replicates of each library were combined and processed as single biological replicate data.

The statistical identification of PIF3-binding peaks was performed separately for each biological replicate using MACS [35] with the default 10^−5^ P-value cutoff. MACS analysis was customized to ensure a more uniform analysis across biological replicates, and to decrease the size of the window for detecting background enrichment (due to the small size of the *Arabidopsis* genome) by employing modified parameters (gsize = 1.1e8, bw = 100, nomodel, shiftsize = 50, slocal = 1000, and llocal = 2000).

Four independent biological replicates of ChIP-seq data were collected, and replicate-specific binding peaks, identified in at least one other replicate, were defined as reproducible, if the distance between the summits of each replicate were less than 100 bp. For each reproducible peak, a mean summit position was assigned as the average position of the individual replicate-specific summits, and the PIF3-binding sites were defined as the 201 bp windows centered at each reproducible mean-summit position.

*De novo* PIF3-binding motif discovery was performed on the 201-bp defined binding sites using MEME [36], and the enrichment significance of identified G-box and PBE-box motifs beyond the genome background was quantified by 100 random simulations, where in each simulation 1064 randomly selected genomic regions of the same size were searched for the occurrence of each motif. The tight association of PIF3 binding with a specific motif was defined as the distance between the peak summit and the closest motif less than 100 bp.

Definition of the closest neighboring genes to each binding peak was approached by scanning the regions within +/−5 kb centered at each peak summit, using CisGenome [51], and the potential target genes downstream of each summit with no intervening genes were selected manually.

### RNA–seq

Total RNA was extracted from 2-d-old dark-grown seedlings using QIAshredder column and RNeasy Plus Mini Kit (Qiagen) according to the manufacturer's instructions. The sequencing library construction was adapted from 3′-end RNA-seq protocol [52]. The mRNA was fractionated from 20 µg of total RNA using Dynabeads Oligo (dT)_25_ (Invitrogen), and fragmented using Fragmentation Reagents (Ambion) at 70°C for 2.5 min in 20 µl of reaction. The polyA-tailed 3′-end fragments were captured by another run of mRNA purification as described above, and then treated by Antarctic Phosphatase (NEB) and T4 Polynucleotide Kinase (NEB) at 37°C for 1 h and 2 h, respectively. The sample was purified using RNeasy MinElute Cleanup Kit (Qiagen) according to Illumina's protocol. The eluted mRNA fragments were ligated with 2.5 µM of Illumina's SRA 5′ adaptor by T4 RNA Ligase 1 (NEB) at 20°C for 4 h. The 3′ cDNA adapter derived from Illumina's v1.5 sRNA 3′ adapter was conjugated with the anchored oligo (dT)_20_ primer, and introduced through reverse transcription using the SuperScript III First-Strand Synthesis System (Invitrogen). The first-strand cDNA was purified using the Agencourt AMPure XP system, and then amplified by PCR reaction using Phusion High-Fidelity DNA Polymerase with Illumina's sRNA PCR primer set. The size of purified library DNA was validated by Bioanalyzer 2000.

Libraries from the 1st biological replicate were assayed by 36-cycle single-end sequencing on the Illumina GAIIx platform, while libraries from the 2nd and 3rd biological replicates were assayed by 50-cycle single-end sequencing on the HiSeq2000 platform. For consistency, only the 5′-end 36-nt trimmed reads from the 2nd and 3rd replicates, as well as the full-length 36-nt reads from the 1st replicate, were aligned to the TAIR9 representative transcriptome using Bowtie with zero mismatches allowed. Only reads mapping uniquely to the 3′-end 500-bp region of the coding strand were counted for gene expression. Differentially expressed genes were identified using the edgeR package [53], and SSTF genes were defined as those that differ by ≥2-fold with an adjusted P value ≤0.05 as described [17].

### *In vitro* protein–DNA binding assay

The recombinant protein GST-PIF3-Flag and the GST control were purified from *E. coli* as described previously [9]. DNA probes were generated by annealing a 5′ biotinylated oligonucleotide (IDT) to a complementary unmodified oligonucleotide (IDT). The complementary oligonucleotides were diluted in annealing buffer (10 mM Tris-HCl (pH 7.5), 50 mM NaCl, 1 mM EDTA) to a final concentration of 40 µM, heated to 95°C for 5 min, and cooled down slowly (0.1°C/second) to 12°C. The same procedure was followed to generate unmodified dsDNA fragments for competition assays. Probes are listed in Table S13.

The DPI-ELISA assays were performed as described [38]. Biotinylated probes were bound to Reacti-Bind Streptavidin High Binding Capacity Coated 96-Well Plates (Thermo Scientific) by applying 2 pmol/well of the probes in TBS-T buffer (20 mM Tris-HCl (pH 7.5), 180 mM NaCl, 0.1% (v/v) Tween 20) for 1 h at 37°C. The wells were blocked with 5% (w/v) non-fat dry milk in TBS-T buffer for 30 min, and then incubated with 100 ng of GST-PIF3-Flag or GST for 1 h. For competition assays, 2, 10 or 50 pmol/well of the unlabeled probes were added at the same time with the proteins. After incubation, the wells were washed 3 times with TBS-T/PBS-T buffer, and then were incubated with 1∶2000 diluted THE GST Antibody [HRP] (GenScript, A00866) in PBS-T buffer for 1 h. The wells were then washed twice with PBS-T and PBS buffers, respectively, after incubation. The protein binding was detected by adding the OPD solution (Thermo Scientific), and the reaction was stopped by 2.5 M sulfuric acid. The color extinction was measured at 490 nm, using 650 nm as a reference wavelength in the ELISA reader.

The EMSA assays were performed as described [9]. 100 ng of recombinant proteins and the biotinylated DNA probes were used in each assay. Gel electrophoresis using native 5% PAGE gel in ice cold 0.5× TBE buffer (280 V, 15 min) was followed by wet-transfer electro blotting to Biodyne B Nylon membrane (Pierce) in 0.5× TBE buffer (80 V, 1 h). The Lightshift Chemiluminescent DNA EMSA kit (Pierce) was used for detection of the biotinylated probes according to the manufacturer's instructions.

### Quantitative PCR (qPCR)

RT-qPCR was performed as described [17]. Each PCR reaction was repeated at least twice, and the mean value of technical replicates was recorded for each biological replicate. Data from biological triplicates were collected, and the mean value with standard error is represented in the bar graphs. Primers and gene accession numbers are listed in Table S13.

### Construction of *pPIL1:LUC* and *pPIF:GUS* transgenic plants

The 1.8 kb *PIL1* promoter region (*pPIL1*) upstream of the ATG was amplified by PCR using the pPIL1-F1/R1 primer set, and then the *Xho*I/*Eco*RI fragment was cloned into the pBluescript II SK (pBSK) vector (Stratagene) to produce pBSK-pPIL1. The G-box mutations were introduced by two-step PCR amplification (using pPIL1-F7/R7, pPIL1-F8/R8, pPIL1-F9/R9, and pPIL1-F10/R10 primer sets), and the *Xho*I/*Mfe*I fragment from the pPIL1-F1/R5 primer set was cloned to replace the unmutated fragment of pBSK-pPIL1 to produce pBSK-mpPIL1. The *Hind*III/*Bam*HI fragment containing the omega-LUC+-rbcS terminator from the pENTR/D-TOPO\arGIp::LUC+ construct was cloned into pBSK-pPIL1 and pBSK-mpPIL1, respectively, to produce pBSK-pPIL1:LUC and pBSK-mpPIL1:LUC. The CDS of Renilla Luciferase (*RLUC*) was amplified by PCR using the Rluc-F1/R1 primer set, and then the *Nco*I/*Pml*I fragment was cloned into the pCAMBIA1302 binary vector to produce pC1302-35S:RLUC. The *Pst*I/*Sac*I fragments from pBSK-pPIL1:LUC and pBSK-mpPIL1:LUC were then sub-cloned into pC1302-35S:RLUC to produce pC1302-pPIL1:LUC-35S:RLUC (*pPIL1:LUC*) and pC1302-mpPIL1:LUC-35S:RLUC (*mpPIL1:LUC*), respectively.

The 2.5–3.0 kb promoter regions upstream of the ATG of *PIF3*, *PIF4* and *PIF5* were amplified from *Arabidopsis* (Col-0 ecotype) genomic DNA by PCR using the TOPO-PIF3p-LP1/RP1, TOPO-PIF4p-LP1/RP1 and TOPO-PIF5p-LP1/RP1 primer sets, respectively. The PCR products were cloned into the pENTR/D-TOPO vector (Invitrogen) to produce the pPIF3, pPIF4 and pPIF5 entry clones. For the *PIF1* promoter, the first 2 kb fragment upstream of the ATG was amplified by PCR using the TOPO-PIF1p-LP3/RP1 primer set, and then was cloned into the pENTR/D-TOPO vector to produce the intermediate entry clone. The second fragment of 2–4 kb upstream of ATG was amplified using the NotI-PIF1p-LP/XcmI-PIF1p-RP primer set, and then the *Not*I/*Xcm*I fragment of the PCR product was subcloned into the intermediate entry clone to produce the pPIF1 entry clone. All four entry clones were subcloned into the gateway compatible pGWB3 binary vector [54] using Gateway LR Clonase II Enzyme Mix (Invitrogen) to produce *pPIF:GUS* constructs.

The constructs were transformed into *Arabidopsis* plants as described [55], and the individual transgenic lines were selected on MS medium containing 25 mg/L of Hygromycin B (Invitrogen).

### Luciferase assay

The 2-d dark-grown seedlings of independent transgenic lines were ground in liquid nitrogen, and total protein was extracted in LUC extraction buffer (1× PBS, 4 mM EDTA, 2 mM DTT, 5% glycerol, 1 mg/ml BSA, 2 mM PMSF and 1× complete protease inhibitor cocktail (Roche) at 3× w/v) as described [7]. 20 µl of the supernatant were used to measure the LUC and RLUC activity using a Dual-Luciferase Reporter Assay System (Promega) according to the manufacturer's instruction. The relative expression of *LUC* was represented by its enzyme activity compared to the RLUC internal control.

### Histochemical GUS staining

Histochemical GUS staining assays were performed on 2-d-old dark-grown seedlings as described [56] using a modified substrate buffer (1× PBS (pH 7.0), 1 mM K_3_Fe(III)(CN)_6_, 0.5 mM K_4_Fe(II)(CN)_6_, 1 mM EDTA, 1% Triton X-100, 1 mg/ml X-gluc). Data of biological triplicates were collected from two independent transgenic lines, and representative images are shown for each transgene.

### Accession number

ChIP-seq and RNA-seq data reported in this study have been deposited in the Gene Expression Omnibus database under the accession number GSE39217.

## Supporting Information

Figure S1 ChIP-qPCR validation of PIF3-binding sites. (A–C) PIF3-binding sites containing the G-box (A), PBE-box (B), or both motifs (C) were validated using independent ChIP-qPCR tests. Sites are named according to adjacent genes. The relative enrichment level is presented as the percentage of input-control DNA that is immunoprecipitated for the P3M and WT samples. Data are presented as the mean of biological triplicates +/− SEM.
(TIF)

Figure S2 EMSA assays of PIF3 *in vitro* binding to the G-box and PBE-box motifs. EMSA assays show that the recombinant GST-PIF3 protein (Lane 3), but not GST by itself (Lane 2), specifically binds to the wild-type (WT) biotinylated G-box and PBE-box probes. For probes containing single G-box or PBE-box motif (*PIL1a*, *PHYB*, *IAA2*, *IBH1* and *AT4G30410*), the cold competitor probes mutated at the motif (mut; Lane 5) did not exhibit competitive effects similar to the WT competitor (Lane 4). For probes containing two G-box motifs (*PIL1b* and *RGA1*), the mut probes at either one motif (Land 6 and 7) showed competitive effects similar to the WT probes (Lane 4), whereas the mut probe at both motifs (Lane 5) did not show efficient competition. The *ATHB-2* probe contains one G-box and one PBE-box motif separated by 5 bp. The mut probe at only the G-box (Lane 6) displayed no competition, as did the probe mutated at both G-box and PBE-box motifs (Lane 5), while the mut probe at only PBE-box motif (Lane 7) showed a competitive effect similar to the WT probe (Lane 4). ^1^ both G-boxes mutated, ^2^ only 1st G-Box mutated, ^3^ only 2nd G-Box mutated, ^4^ G-box and PBE-box mutated, ^5^ only 1st PBE-box mutated.
(TIF)

Figure S3 RT-qPCR validation of PIF3- and PIF-quartet-regulated genes. (A and B) The orange horizontal bar graphs show the relative PIF3-induced expression of 19 Z-Class (A) and 88 Y-Class (B) PIF-induced genes as determined by RNA-seq analysis. The relative PIF3-induced expression value was calculated as the ratio of the level in the *pif145* mutant compared to that in the *pifq* mutant (*pif145/pifq*), and the genes are arrayed according to this ratio. We assayed the expression patterns of 12 (A) and 20 (B) genes in each class using RT-qPCR, and the expression relative to WT was normalized to the *PP2AA3* internal control, for the *pif3*, *pif145* and *pifq* mutants. The data are presented as the mean of biological triplicates +/− SEM in the vertical bar-graph boxes, compared to the RNA-seq data for these genes. Genes highlighted-in-red exhibited statistically-significant differences between *pif145* and *pifq* in the RT-qPCR tests, by Student's *t*-test (*P*<0.05) (indicated by red star above the *pif145* bar), while genes highlighted-in-blue did not. Data from RNA-seq are shown as the fold-change determined by the edgeR package in each *pif* mutant compared to WT. The internal percentage values in each box represent the relative contribution of PIF3 to the total PIF-quartet-induced transcription for each tested gene, as shown in Figure 5.
(TIF)

Figure S4 Relative contribution of PIF3 and the PIF1/4/5-trio to the collective regulation of the PIF quartet. The relative contribution of PIF3 and the PIF1/4/5-trio to the collective regulation of the PIF quartet is plotted for the 107 PIF3-bound, PIF-induced genes (Classes Y and Z). The degree of collective regulation by the PIF quartet is defined as the difference in expression between *pifq* and WT. The separate activities of PIF3 and the combined PIF1/4/5-trio, are defined as the differences in expression between *pif145* and *pifq*, and between *pif145* and WT, respectively. The relative contribution is then defined as the percentage of the collective PIF-quartet regulation contributed by the separate activities of PIF3 and the combined PIF1/4/5-trio, respectively.
(TIF)

Figure S5 Functional categorization of PIF3-bound, PIF-regulated genes. (A) The total PIF3-bound, PIF-regulated genes identified by integrating our ChIP-seq and RNA-seq analyses (Classes X, Y and Z) were assigned to functional categories, color-coded as shown. This assignment was based on the Gene Ontology annotations for biological and/or molecular function in the TAIR database. The percentage of the total annotated genes within each category was calculated after excluding the genes annotated as having unknown function. (B) PIF-induced and PIF-repressed genes in Classes X, Y and Z were assigned separately to the functional categories as described in (A). The first numbers in parentheses represent the genes with functional annotation, and the second numbers represent the total genes in each class.
(TIF)

Figure S6 Correlated PIF3-binding and PIF-regulation of eight M-class genes. (A–H) Visualization of ChIP-seq and RNA-seq data in the genomic regions encompassing *TCP15* (A), *IAA29* (B), *ATHB52* (C), *CKX5* (D), *XTR7* (E), *SNRK2.5* (F), *AT5G02580* (G), *and SDR* (H), respectively. The ChIP and RNA tracks show the pile-up distribution of the combined raw reads from four biological replicates of ChIP-seq data and three replicates of RNA-seq data, respectively. P3M- and WT-ChIP: DNA immunoprecipitated from PIF3-Myc-expressing and from wild-type seedlings, respectively. WT-, *pif3-*, *pif145-* and *pifq*-RNA: RNA from genotypes used for expression analysis. Binding Site: 201 bp defined as the PIF3-binding site. Summit, predicted PIF3-binding center defined from the binding-peak maximum. G- and PBE-box: Vertical lines indicate motif positions.
(TIF)

Figure S7 Comparison of potential direct targets of PIF3, PIF4 and/or PIF5. (A) Venn diagram shows overlap of genes associated with PIF3, PIF4 and/or PIF5 binding sites. (B) Venn diagram shows PIF-quartet-regulated genes bound by PIF3, PIF4 and/or PIF5.
(TIF)

Table S1 List of PIF3-binding peaks from four ChIP-seq replicates. (A) List of PIF3-binding peaks from the 1st replicate. (B) List of PIF3-binding peaks from the 2nd replicate. (C) List of PIF3-binding peaks from the 3rd replicate. (D) List of PIF3-binding peaks from the 4th replicate.
(XLS)

Table S2 List of reproducible PIF3-binding sites.
(XLS)

Table S3 List of PIF3-bound genes.
(XLS)

Table S4 List of SSTF genes from *pifq*/WT comparison (PIF-quartet-regulated genes).
(XLS)

Table S5 List of SSTF genes from *pif3*/WT comparison (PIF3-regulated genes (loss-of-function)).
(XLS)

Table S6 List of SSTF genes from *pif145*/WT comparison (PIF1/4/5-trio-regulated genes (loss-of-function)).
(XLS)

Table S7 List of SSTF genes from *pifq*/*pif145* comparison (PIF3-regulated genes (gain-of-function)).
(XLS)

Table S8 List of SSTF genes from *pifq*/*pif3* comparison (PIF1/4/5-trio-regulated genes (gain-of-function)).
(XLS)

Table S9 Composite list of PIF3-regulated genes.
(XLS)

Table S10 Composite list of PIF1/4/5-trio-regulated genes.
(XLS)

Table S11 List of PIF3-, PIF4 and/or PIF5-bound, PIF-regulated (Class X, Y and Z) genes.
(XLS)

Table S12 List of rapidly-light-regulated W-class genes.
(XLS)

Table S13 List of oligonucleotides used in this study. (A) List of ChIP-qPCR primers. (B) List of RT-qPCR primers. (C) List of cloning primers. (D) List of *in vitro* binding probes.
(XLS)
