# Supplementary figures and images for: A Quartet of PIF bHLH Factors Provides a Transcriptionally Centered Signaling Hub That Regulates Seedling Morphogenesis through Differential Expression-Patterning of Shared Target Genes in Arabidopsis
Source: PLoS Genet. 2013 Jan 31;9(1):e1003244. doi: 10.1371/journal.pgen.1003244 (PMC3561105; doi:10.1371/journal.pgen.1003244)

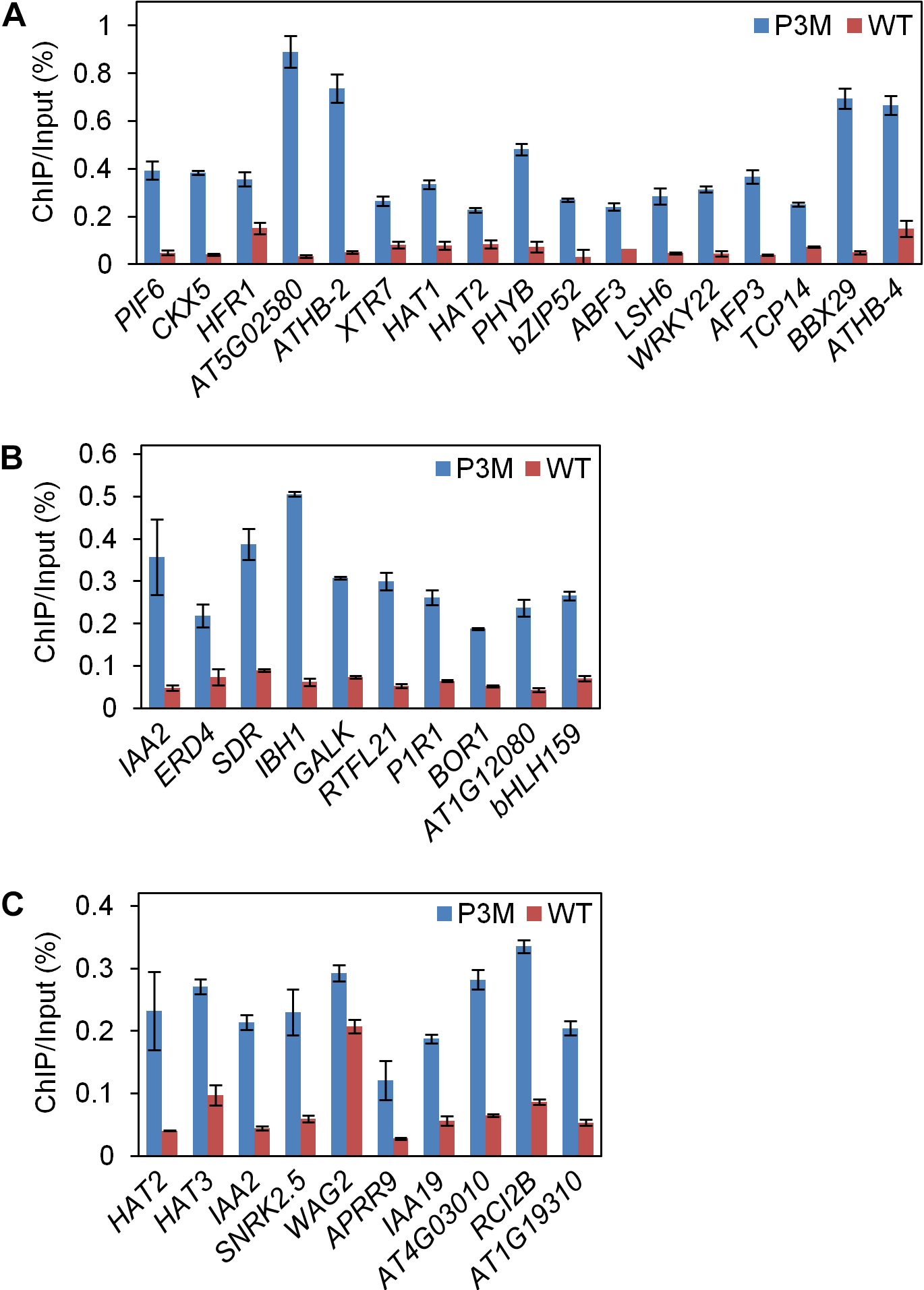

Supplement: Figure S1 — ChIP-qPCR validation of PIF3-binding sites. (A–C) PIF3-binding sites containing the G-box (A), PBE-box (B), or both motifs (C) were validated using independent ChIP-qPCR tests. Sites are named according to adjacent genes. The relative enrichment level is presented as the percentage of input-control DNA that is immunoprecipitated for the P3M and WT samples. Data are presented as the mean of biological triplicates +/− SEM. (TIF) [file pgen.1003244.s001.tif]

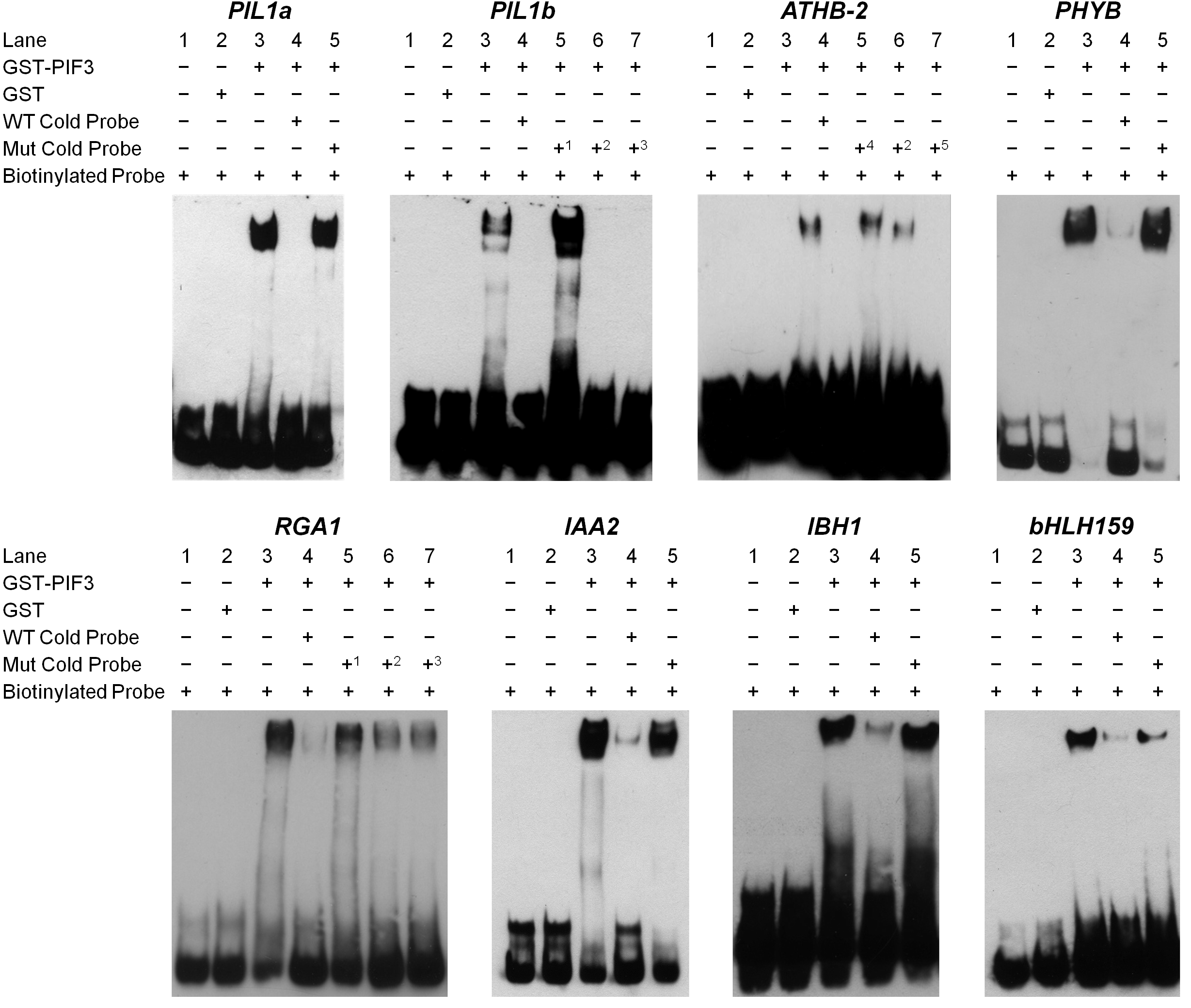

Supplement: Figure S2 — EMSA assays of PIF3 in vitro binding to the G-box and PBE-box motifs. EMSA assays show that the recombinant GST-PIF3 protein (Lane 3), but not GST by itself (Lane 2), specifically binds to the wild-type (WT) biotinylated G-box and PBE-box probes. For probes containing single G-box or PBE-box motif (PIL1a, PHYB, IAA2, IBH1 and AT4G30410), the cold competitor probes mutated at the motif (mut; Lane 5) did not exhibit competitive effects similar to the WT competitor (Lane 4). For probes containing two G-box motifs (PIL1b and RGA1), the mut probes at either one motif (Land 6 and 7) showed competitive effects similar to the WT probes (Lane 4), whereas the mut probe at both motifs (Lane 5) did not show efficient competition. The ATHB-2 probe contains one G-box and one PBE-box motif separated by 5 bp. The mut probe at only the G-box (Lane 6) displayed no competition, as did the probe mutated at both G-box and PBE-box motifs (Lane 5), while the mut probe at only PBE-box motif (Lane 7) showed a competitive effect similar to the WT probe (Lane 4). 1 both G-boxes mutated, 2 only 1st G-Box mutated, 3 only 2nd G-Box mutated, 4 G-box and PBE-box mutated, 5 only 1st PBE-box mutated. (TIF) [file pgen.1003244.s002.tif]

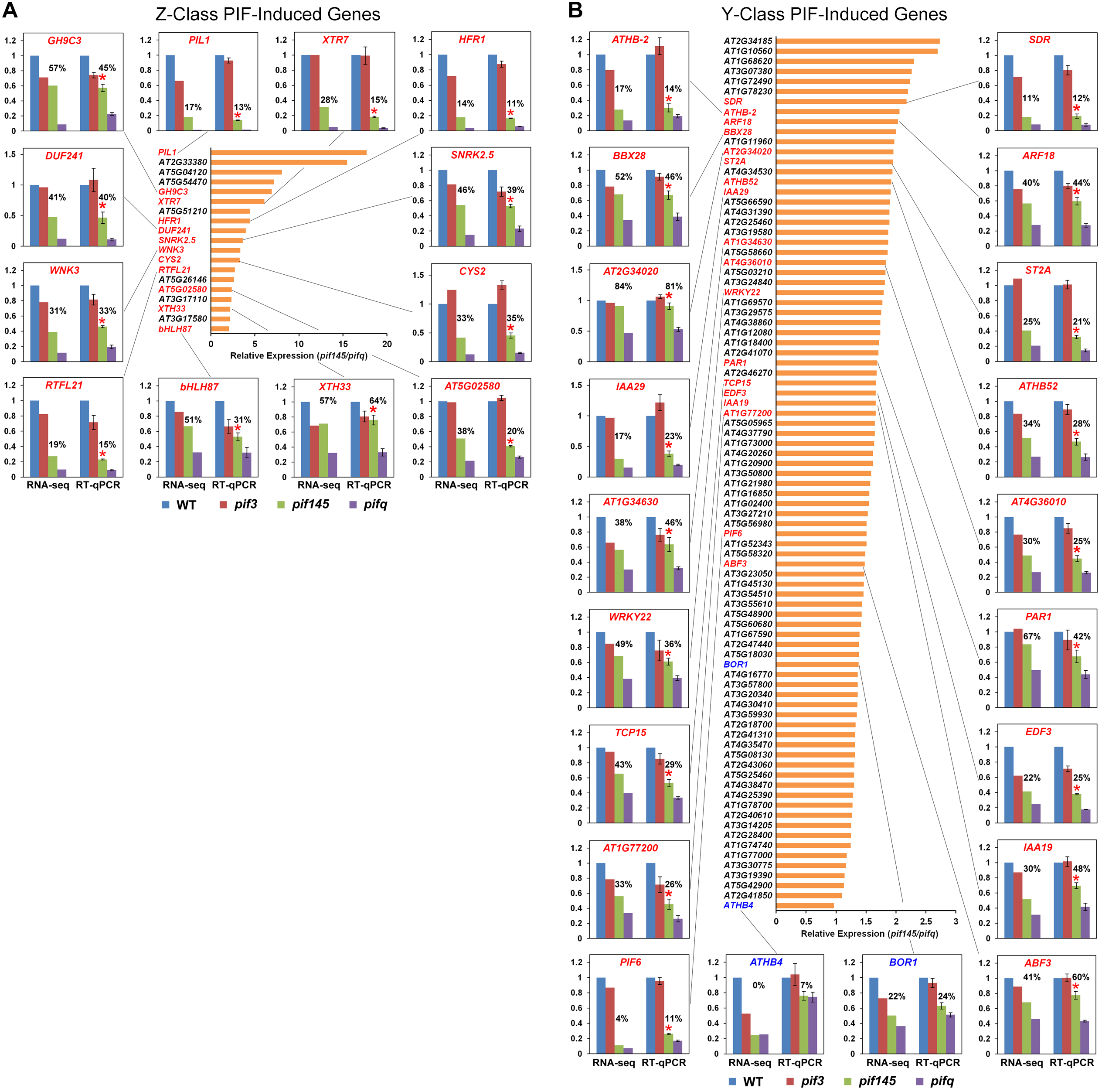

Supplement: Figure S3 — RT-qPCR validation of PIF3- and PIF-quartet-regulated genes. (A and B) The orange horizontal bar graphs show the relative PIF3-induced expression of 19 Z-Class (A) and 88 Y-Class (B) PIF-induced genes as determined by RNA-seq analysis. The relative PIF3-induced expression value was calculated as the ratio of the level in the pif145 mutant compared to that in the pifq mutant (pif145/pifq), and the genes are arrayed according to this ratio. We assayed the expression patterns of 12 (A) and 20 (B) genes in each class using RT-qPCR, and the expression relative to WT was normalized to the PP2AA3 internal control, for the pif3, pif145 and pifq mutants. The data are presented as the mean of biological triplicates +/− SEM in the vertical bar-graph boxes, compared to the RNA-seq data for these genes. Genes highlighted-in-red exhibited statistically-significant differences between pif145 and pifq in the RT-qPCR tests, by Student's t-test (P<0.05) (indicated by red star above the pif145 bar), while genes highlighted-in-blue did not. Data from RNA-seq are shown as the fold-change determined by the edgeR package in each pif mutant compared to WT. The internal percentage values in each box represent the relative contribution of PIF3 to the total PIF-quartet-induced transcription for each tested gene, as shown in Figure 5. (TIF) [file pgen.1003244.s003.tif]

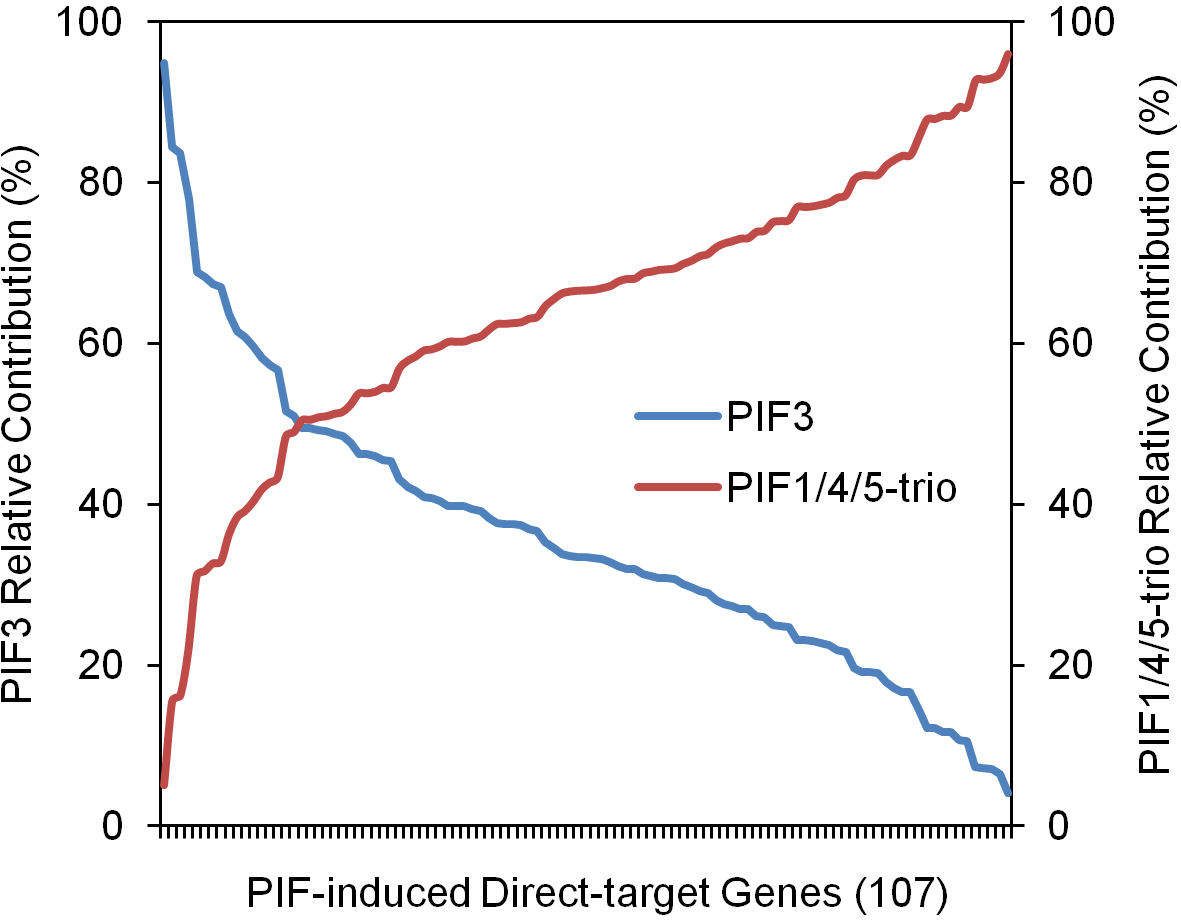

Supplement: Figure S4 — Relative contribution of PIF3 and the PIF1/4/5-trio to the collective regulation of the PIF quartet. The relative contribution of PIF3 and the PIF1/4/5-trio to the collective regulation of the PIF quartet is plotted for the 107 PIF3-bound, PIF-induced genes (Classes Y and Z). The degree of collective regulation by the PIF quartet is defined as the difference in expression between pifq and WT. The separate activities of PIF3 and the combined PIF1/4/5-trio, are defined as the differences in expression between pif145 and pifq, and between pif145 and WT, respectively. The relative contribution is then defined as the percentage of the collective PIF-quartet regulation contributed by the separate activities of PIF3 and the combined PIF1/4/5-trio, respectively. (TIF) [file pgen.1003244.s004.tif]

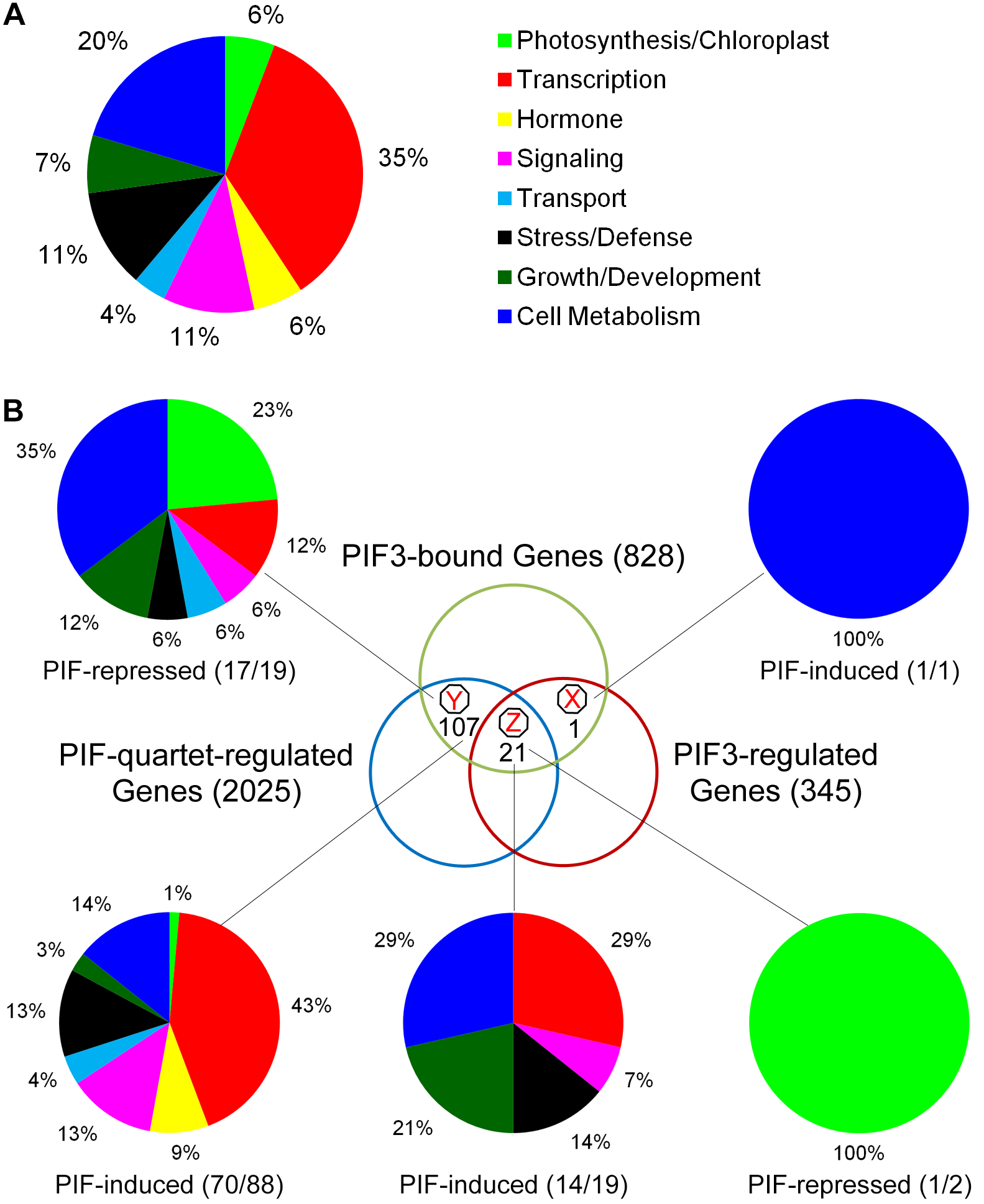

Supplement: Figure S5 — Functional categorization of PIF3-bound, PIF-regulated genes. (A) The total PIF3-bound, PIF-regulated genes identified by integrating our ChIP-seq and RNA-seq analyses (Classes X, Y and Z) were assigned to functional categories, color-coded as shown. This assignment was based on the Gene Ontology annotations for biological and/or molecular function in the TAIR database. The percentage of the total annotated genes within each category was calculated after excluding the genes annotated as having unknown function. (B) PIF-induced and PIF-repressed genes in Classes X, Y and Z were assigned separately to the functional categories as described in (A). The first numbers in parentheses represent the genes with functional annotation, and the second numbers represent the total genes in each class. (TIF) [file pgen.1003244.s005.tif]

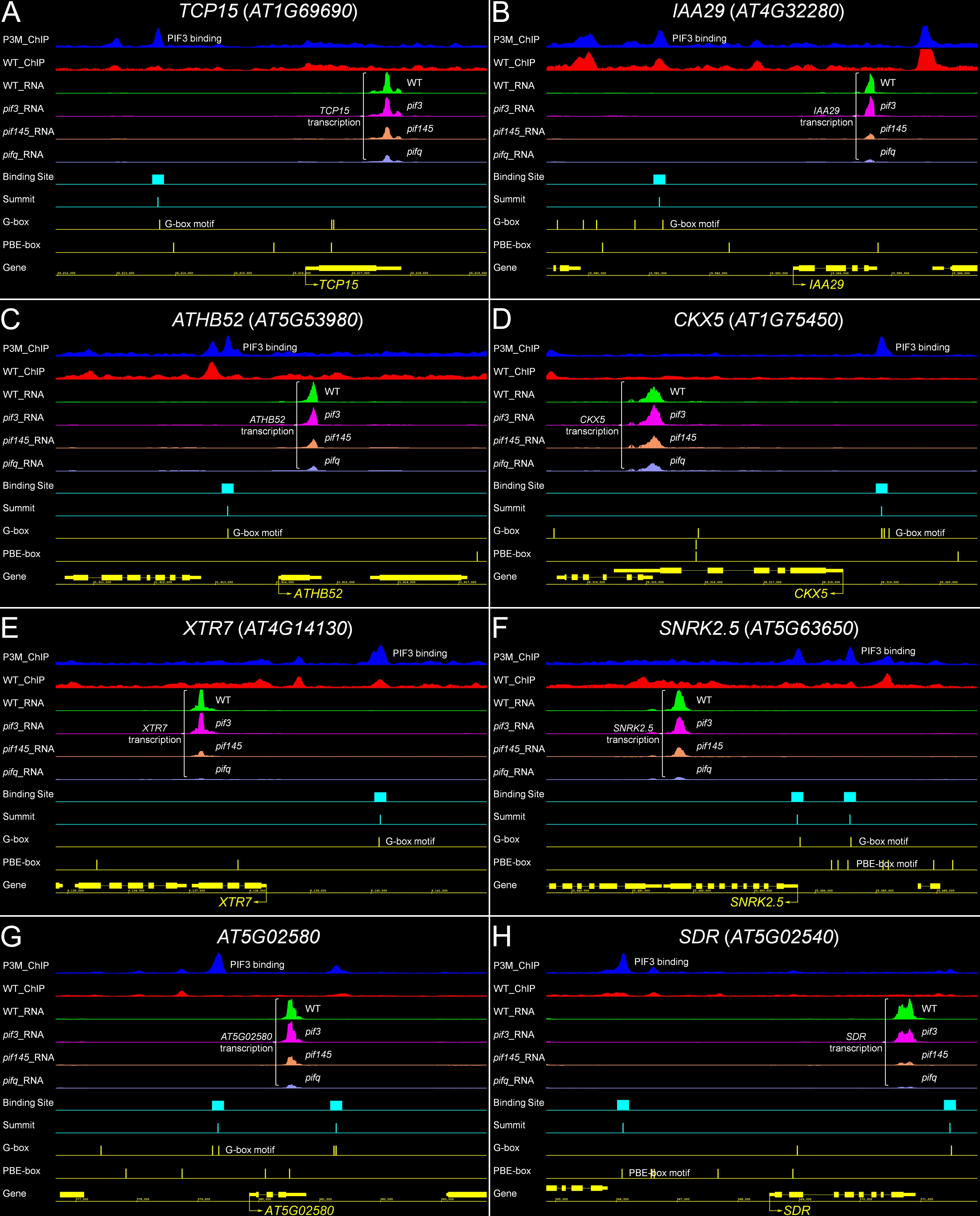

Supplement: Figure S6 — Correlated PIF3-binding and PIF-regulation of eight M-class genes. (A–H) Visualization of ChIP-seq and RNA-seq data in the genomic regions encompassing TCP15 (A), IAA29 (B), ATHB52 (C), CKX5 (D), XTR7 (E), SNRK2.5 (F), AT5G02580 (G), and SDR (H), respectively. The ChIP and RNA tracks show the pile-up distribution of the combined raw reads from four biological replicates of ChIP-seq data and three replicates of RNA-seq data, respectively. P3M- and WT-ChIP: DNA immunoprecipitated from PIF3-Myc-expressing and from wild-type seedlings, respectively. WT-, pif3-, pif145- and pifq-RNA: RNA from genotypes used for expression analysis. Binding Site: 201 bp defined as the PIF3-binding site. Summit, predicted PIF3-binding center defined from the binding-peak maximum. G- and PBE-box: Vertical lines indicate motif positions. (TIF) [file pgen.1003244.s006.tif]

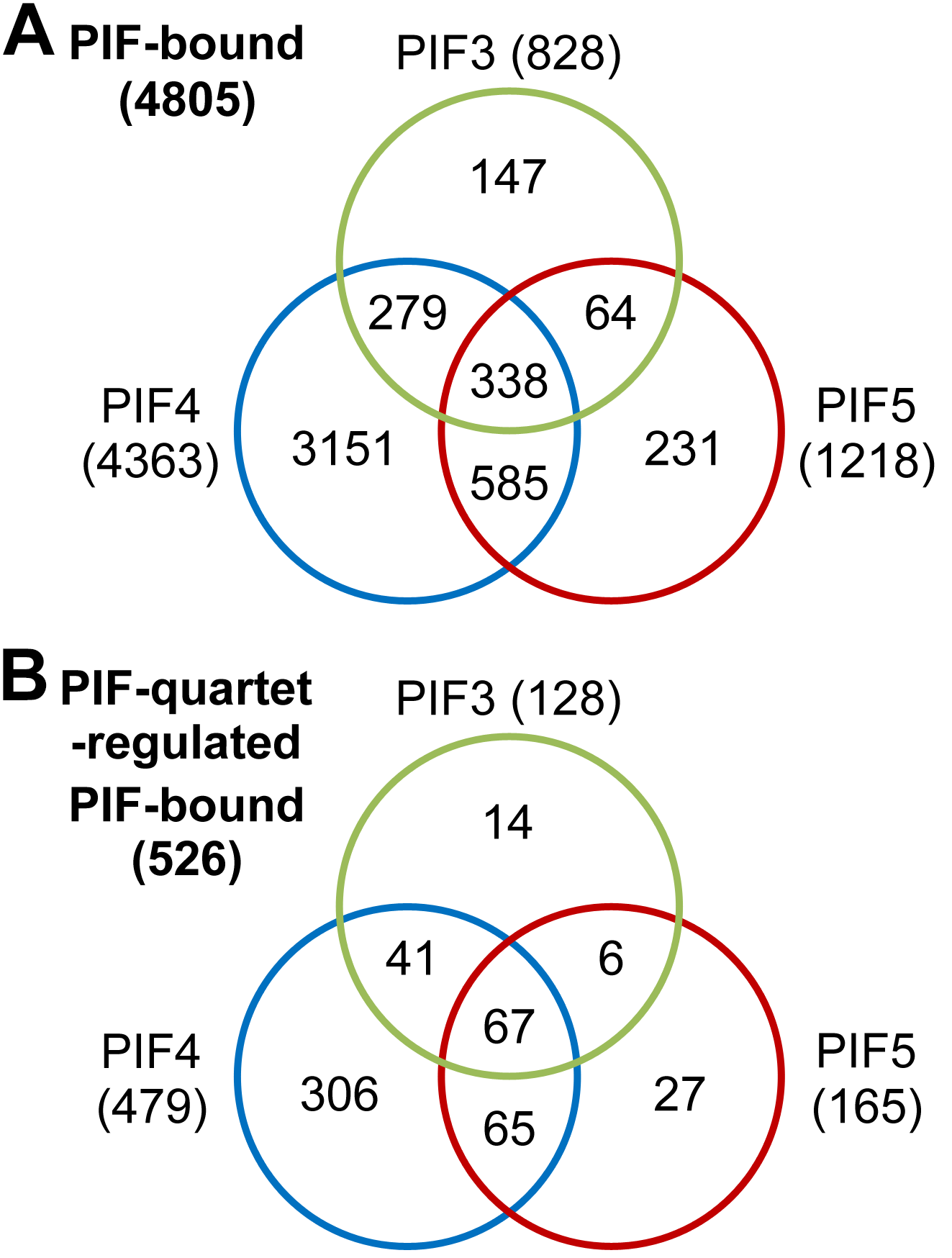

Supplement: Figure S7 — Comparison of potential direct targets of PIF3, PIF4 and/or PIF5. (A) Venn diagram shows overlap of genes associated with PIF3, PIF4 and/or PIF5 binding sites. (B) Venn diagram shows PIF-quartet-regulated genes bound by PIF3, PIF4 and/or PIF5. (TIF) [file pgen.1003244.s007.tif]
